# Drug Delivery Systems Repurposing Taxanes as Antitumor Immune Modulators

**DOI:** 10.34133/bmr.0390

**Published:** 2026-07-09

**Authors:** Sang-Hun Choi, Soo-Hyang Chi, Chae Yeon Han, Kyeong Jin Cho, Sejin Son, Susan N. Thomas, Jihoon Kim

**Affiliations:** ^1^ School of Integrative Engineering, Chung-Ang University, Seoul 06974, Republic of Korea.; ^2^ Department of Biological Sciences, Inha University, Incheon 22212, Republic of Korea.; ^3^ George W. Woodruff School of Mechanical Engineering, Georgia Institute of Technology, Atlanta 30332, GA, USA.; ^4^ Parker H. Petit Institute for Bioengineering and Bioscience, Georgia Institute of Technology, Atlanta 30332, GA, USA.; ^5^ Wallace H. Coulter Department of Biomedical Engineering, Georgia Institute of Technology and Emory University, Atlanta 30332, GA, USA.; ^6^ Winship Cancer Institute, Emory University, Atlanta 30322, GA, USA.

## Abstract

Taxane chemotherapeutics, including paclitaxel, docetaxel, and cabazitaxel, are commonly used in cancer therapy because of their cytotoxic effects against malignant cells. Although their primary mechanisms involve microtubule stabilization and induction of apoptosis, emerging evidence suggests that taxanes also modulate antitumor immunity. Drug delivery systems (DDSs) have been used to enhance taxane therapeutic efficacy by improving tumor targeting and immune modulation. This review discusses the immunomodulatory effects of taxanes and their effects on spatially distinct immune processes within tumors and draining lymph nodes. We highlight recent advances in taxane DDSs that facilitate tumor- and lymphatic-targeted delivery, thereby enhancing antigen presentation, adaptive immune activation, and systemic antitumor immunity. Furthermore, we explore synergistic strategies that combined taxane-based DDSs with immune checkpoint inhibitors and other immunotherapeutic agents. By integrating these approaches, taxane-based chemoimmunotherapy presents potential for advancing next-generation cancer immunotherapy.

## Introduction

**Fig. 1.**
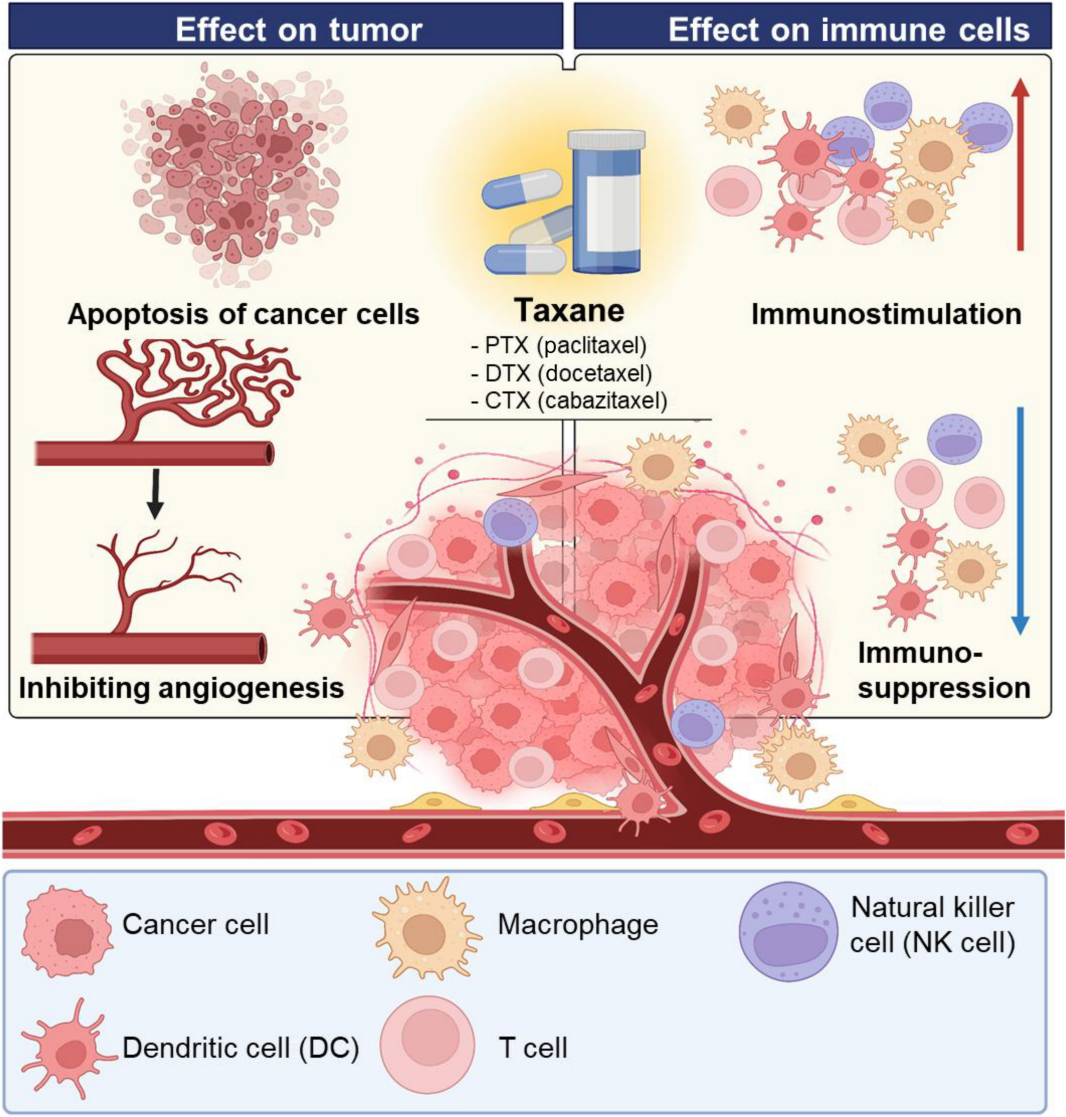
Overview of the role of taxane in cancer cells and immunity. The illustration was created using BioRender.com.

Taxanes are a class of antimicrotubular agents that include paclitaxel (PTX), docetaxel (DTX), and cabazitaxel (CTX), all of which are used clinically [1]. PTX and DTX were first isolated from Pacific Yew *Taxus brevifolia* and European Yew *T. baccata*, respectively, whereas CTX is a semisynthetic taxoid derived from 10-deacetylbaccatin III [1]. PTX, DTX, and CTX exert antitumor effects by inducing cancer cell apoptosis [1,2] and inhibiting angiogenesis [1–4] and have been widely used to successfully treat various tumors, including ovarian, breast, head, neck, lung, and prostate cancers, in the clinic for several decades (Fig. 1). However, inherited and acquired resistance limits taxane therapeutic effects and even result in resistance to other types of anticancer drugs by altering drug efflux and metabolism and inducing mutations in drug targets [5–7].

Drug delivery systems (DDSs) improve the bioavailability and pharmacokinetics/pharmacodynamics of drugs and alter the drug uptake mechanisms that affect drug efflux and metabolism, which reduce side effects and enhance the therapeutic effects of drugs [8,9]. To improve the therapeutic index of taxanes [10,11], various DDSs utilizing polymer–drug conjugates [12], nanoparticles (NPs) [13–17], microparticles [18], hydrogels [19,20], and microneedles [21] have been developed. Such developments have led to the clinical use of *nab*-PTX (Abraxane), which is composed of PTX and albumin [22–26]. Nevertheless, taxanes (such as Abraxane) show low response rate (<25%) in patients, which occasionally incur tumor relapse, even after treatment [27–29].

With recent highlights and advances in cancer immunotherapy, represented by chimeric antigen receptor-T and immune checkpoint blockade (ICB) therapies [30–33] and the widespread clinical use of taxanes in medical oncology in combination with these immunotherapies, an expanded understanding of the complexities of cancer and immune cells in the tumor microenvironment (TME) has led to the reevaluation of the mechanism of action of various chemotherapeutic drugs in clinic [33]. In this regard, taxanes are an interesting class of drugs that manifest paradoxical effects, as shown by their clinical use in anticancer drugs to kill cancer cells [1–4,10–26] and activate immunostimulatory cells [34] and pre(clinical) exploration as immunosuppressive agents for organ transplantation [35]. These seemingly paradoxical uses suggest that the antitumor effects of taxanes are strongly influenced by the immune system in addition to the classical mechanisms associated with direct apoptosis of cancer cells, inhibition of angiogenesis, and drug resistance.

Herein, we discuss the current understanding of the effects of taxanes on the immune response and highlight the efforts to leverage the advantages of DDSs in taxane-mediated chemoimmunotherapy. With sustained attention paid to the benefits of taxane therapies, the rationale for this review is to illuminate their potential to advance smart chemoimmunotherapy approaches, considering the next imminent frontier in medical oncology [33].

## Effects of Taxanes on the Immunogenicity of Cancer Cells In Vitro

Antitumor immunogenicity is the ability of a molecule or substance to provoke an antitumor immune response [36], which results from effects on both cancer and immune cells. Owing to numerous review articles having summarized and discussed the cytotoxic mechanisms of taxanes on cancer cells [1–7], we restrict our discussion to the direct in vitro effects of taxanes on the immunogenicity of cancer cells in this section, followed by those on immune cells in the next section. This fundamental background assists comprehension of the complex immune reactions orchestrated by taxane DDSs in vivo.

### Effects of taxanes on cancer cell immunogenic cell death (ICD)

**Fig. 2.**
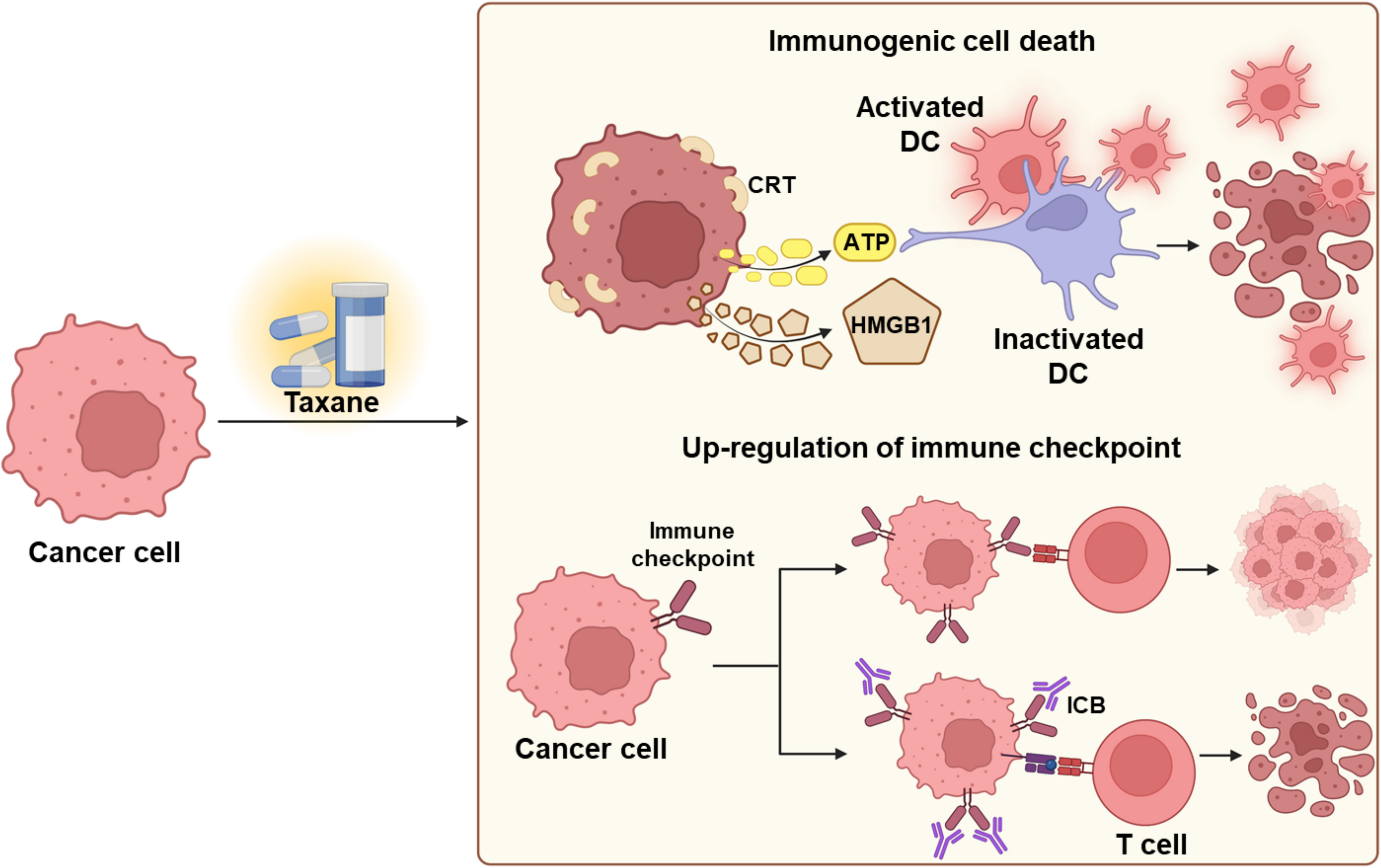
Schematic illustration summarizing the effects of taxanes on cancer cells, particularly immunogenic cell death (ICD) and immune checkpoint blockade (ICB) expression. The illustration was created using BioRender.com.

ICD is a mechanism of antitumor immunogenicity initiated by cancer cells stressed or injured by agents or methodologies that induce cytotoxicity, which causes cancer cells to release, translocate, or surface-expose damage-associated molecular patterns, including adenosine triphosphate (ATP), high-mobility group protein box 1 (HMGB1), and calreticulin (CRT) (Fig. 2) [37]. Notably, not all anticancer drugs can induce ICD; among the 114 chemical drugs approved by the Food and Drug Administration as anticancer agents as of 2014, only 6 drugs, including daunorubicin, doxorubicin, mitoxantrone, oxaliplatin, PTX, and DTX, were validated to elicit 1 or more ICD characteristics [38]. Free PTX and PTX-loaded pH-sensitive micelles substantially triggered CRT, HMGB1, and ATP in B16F10 melanoma cell lines [39]. Similarly, free PTX and PTX-loaded NPs formulated by multivalent host–guest interactions exhibited enhanced extracellular release of ATP and surface expression of CRT upon treatment of B16F10 [40]. PTX-loaded pH- and matrix metalloproteinase-2-sensitive micelles also induced substantial CRT expression in B16F10 [41]. In addition to the B16F10 cell lines, PTX with various DDSs elicits at least 1 ICD marker in various cell lines, including breast cancer (MDA-MB-231 and 4T1), colon cancer (HCT116, MC38, and CT26), and glioblastoma (GL261, G7, WL1, U87, U251, and G422) [41–51]. However, the ICD mechanisms capable of being provoked by PTX are dependent on the cell type. For instance, a NP self-assembled from indocyanine green and PTX failed to induce CRT expression, leading to HMGB1 translocation and ATP release in 4T1 cells [52]. DTX provokes ATP release, HMGB1 release, and surface exposure to CRT in lung carcinoma cells (NCI-H1975, NCI-H1650, A549, 3LL, and SPC-A1) [53,54], but its effects are dependent on the cell type. For instance, DTX treatment led to enhanced CRT expression in MCF-7, MDA-MB-231, prostate cancer cells (LNCaP), and colorectal cancer cells (SW620), whereas HMGB1 and ATP levels were not changed [55,56]. Although CTX was not identified as an ICD-inducing anticancer drug in 2014 [38], recent studies have discovered that CTX can lead to surface exposure to CRT with the release of HMGB1 in a prostate cancer cell line (Tramp-C1) [57] or release of ATP in a 4T1 cell line [58], respectively. Overall, these results indicate that taxane derivatives facilitate the ICD of various cancer cells, not only implying that clinical taxane-mediated chemotherapy has been associated with chemoimmunotherapy although it was not clearly recognized but also emphasizing tumor tissues as a major target for taxanes and taxane-loaded DDSs (Fig. 2). Notably, DDSs render taxanes immunogenic; for example, free DTX failed to elicit ICD from 4T1 cells in vitro, in contrast to that of DTX-loaded NPs [59].

### Effects of taxanes on immune checkpoints and their ligand expression by cancer cells

Immune checkpoints and their ligands are expressed in various immune and cancer cells, leading to immunosuppressive effects [60]. Immune checkpoints and their ligands include programmed cell death protein-1 (PD-1), programmed death-ligand 1 (PD-L1), cytotoxic T-lymphocyte associated protein 4 (CTLA-4), lymphocyte-activation gene 3, T cell immunoglobulin and mucin domain 3 (TIM3), and T cell immunoreceptors with immunoglobulin and ITIM domain (TIGIT) [31,60]. Cancer cells threatened by therapeutic agents exploit immune checkpoints and their ligands to evade immune surveillance in clinical biospecimens [61–63]. Accordingly, the tumor expression of immune checkpoints and their ligands can be used as biomarkers to predict the prognosis and immunotherapy responses of patients [64–66].

The direct effect of taxanes on the expression of various immune checkpoints and their ligands in cancer cells has not been well investigated in vitro, beyond the effects of taxanes on PD-L1 expression. Free PTX markedly increased PD-L1 expression in the breast cancer (MDA-MB-231, MDA-MB-468, and HS578T) and A549 cell lines [63,67,68]. DTX substantially increased PD-L1 expression in breast cancer (MDA-MB-231, HS578T, and SKBR3 cells) and CTLA-4 expression in cervical cancer (CaSki) cell lines [63,68,69]. CTX also led to PD-L1 overexpression on Tramp C1 and 4T1 cell lines in vitro [57,58]. These results correlate with reports using various taxane-loaded NPs including micelles [41,45,70], protein assemblies [58], and liposomes [71], which also exhibited increased PD-L1 expression in various cancer cells following treatment. Taxane-mediated up-regulation of immune checkpoints and their ligands thus clearly supports the rationale for combined taxane and ICB therapy [33], wherein taxanes are expected to exert cytotoxic effects on cancer cells and ICBs to prevent the immune checkpoint-mediated anergy of T cells in the tumor bed [72–74].

## Effects of Taxanes on Immune Cells In vitro

The main paradigm of cancer immunotherapy is to exploit an adaptive immune cycle, mainly comprising dendritic cells (DCs), T cells, and B cells, to efficiently and selectively kill cancer cells in an antigen-specific manner by eliciting humoral and cellular immune responses [75,76]. Cancer immunotherapy engineering the activity and functions of innate immune cells including macrophages (Mφs) and natural killer (NK) cells has been also under extensive development for future clinical translation [77,78]. In addition, immunosuppressive pathways, mainly regulated by regulatory T cells (T_reg_s) and myeloid-derived suppressor cells (MDSCs), are important factors that should be considered in controlling the therapeutic efficacy and safety of cancer immunotherapy [79–82]. Accordingly, the direct in vitro effects of taxanes on major immune cells, including DCs, T cells, B cells, Mφs, NK cells, and MDSCs, which orchestrate the immune responses to taxane-mediated chemoimmunotherapy in vivo, are described [35,83].

### Effects of taxanes on DCs

DCs are professional antigen-presenting cells (APCs) that are likely the most potent in directing adaptive immune responses. DCs are highly phagocytic, though this depends on their maturation state [84], and thus sample and process cancer antigens, interpret and respond to activation/maturation signals, migrate to regional draining lymph nodes (dLNs), and instruct lymphocytes accordingly [75]. As such, DCs sense and take up taxanes directly or by ingesting cancer cells that undergo taxane-mediated apoptosis during taxane treatment.

**Fig. 3.**
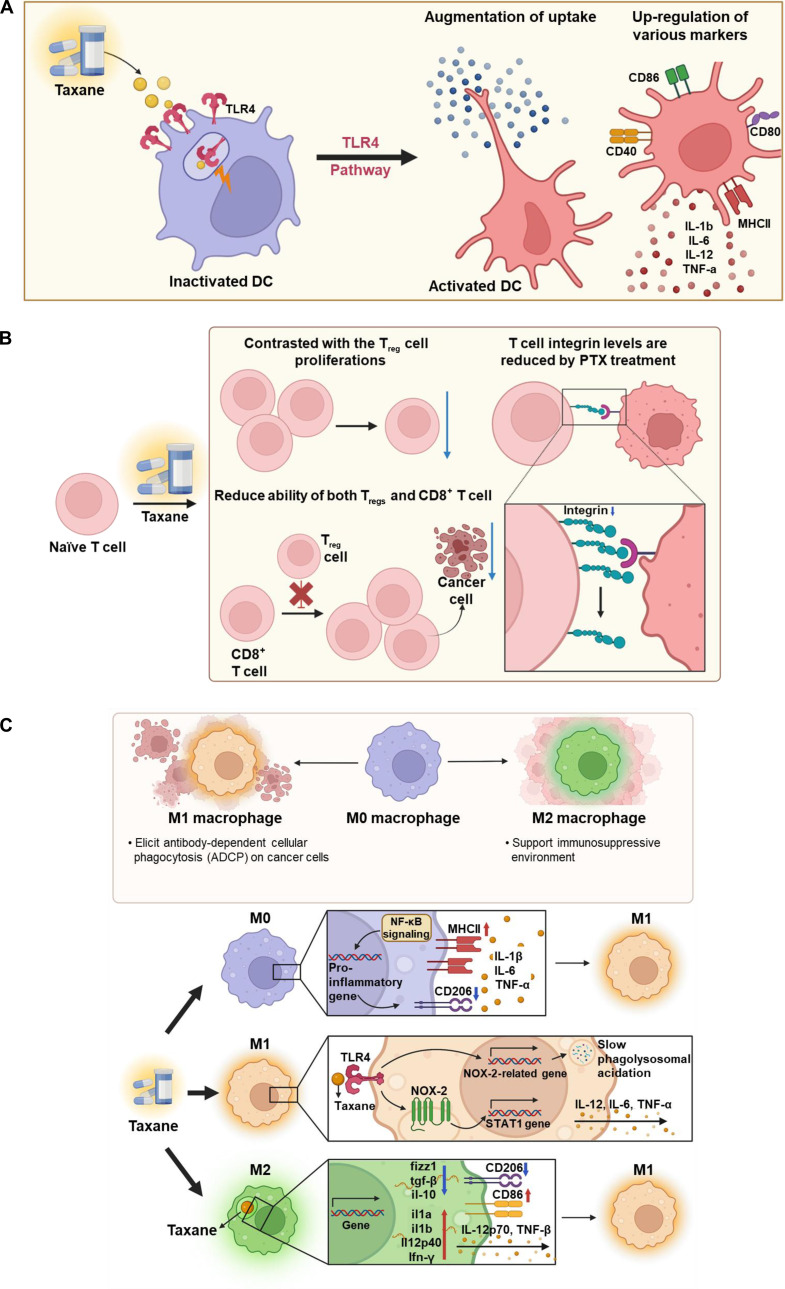
Schematic illustrations of the immunomodulatory effects of taxanes on professional antigen-presenting cells and T cells. (A) Effects of taxanes on dendritic cells (DCs), specifically summarizing their impact on DC uptake ability and Toll-like receptor 4 (TLR4) signaling for maturation and activation. (B) Regulation of T cell-mediated inflammatory immunity, illustrating the mechanisms of functional enhancement or suppression by taxanes. (C) Influence on macrophage (Mφ) polarization and subsequent immune modulation within the microenvironment. All illustrations were created using BioRender.com.

Considering the crucial role of DCs in initiating the adaptive immune cycle, the direct in vitro effects of taxanes on DCs have been extensively explored. PTX considerably augmented the cellular uptake of substances by in vitro bone marrow-dendritic cells (BMDCs), whereas DTX did not alter the uptake ability of DCs [85,86]. In addition, PTX exhibits concentration-dependent (0.1 to 10 𝜇M) DC activation without significant cytotoxicity on the XS106 DC line [85]. PTX also increases the expression of activation markers including cluster of differentiation (CD) 40, CD80, CD86, and major histocompatibility complex II (MHCII) on BMDCs, resulting in the substantial production of interleukin-1𝛽 (IL-1𝛽), IL-6, IL-12, and tumor necrosis factor-𝛼 (TNF-𝛼) [40,46,48,85]. These adjuvant functions of PTX depend on the Toll-like receptor 4 (TLR4) pathways as confirmed by the research demonstrating that an antagonist monoclonal antibody to TLR4 abrogated the PTX-mediated activation of DCs [87]. In contrast with PTX, DTX exhibits limited ability to activate DCs. DTX only leads DCs to substantial increase of IL-1𝛽 [85] and IL-12 [86] with negligible changes in the expression of surface-activation markers [85]. CTX treatment also notably increases CD80 and CD86 expression as well as TNF-𝛼 and IL-12p70 secretion from BMDCs, while decreasing anti-inflammatory IL-10 [57,58]. Despite the different effects, PTX, DTX, and CTX all enhance DC instruction of allogenic T cells, as shown in the increased proliferation of T cells in mixed lymphocyte reactions (MLRs) [57,85,86]. The effects of taxanes may also vary depending on DC activation status; immature DCs treated with PTX exhibit negligible changes in surface activation markers including CD40, CD80, and CD86 and slight increases in IL-12 production, whereas mature DCs treated with PTX increase CD86 and IL-12 expression/production [88]. Various PTX, DTX, and CTX-loaded DDSs also activate DCs and produce proinflammatory cytokines in vitro [40,45–47,58,89], which is in line with results from treatment with free taxanes (Fig. 3A). However, conflicting reports exist on whether taxanes exert proinflammatory or anti-inflammatory effects; significant endocytic function inhibition of DCs and DC-mediated allogenic T cell proliferation have also been reported [90,91]. These conflicts appear to be associated with the different in vitro conditions including the concentration of taxanes and T cells/DC ratios in MLR assays [91]. For instance, in 1 study, 10 𝜇M PTX exhibited the highest efficacy of enhanced DC-mediated allogenic T cell proliferation, compared to that of 1, 100, and 1,000 𝜇M PTX [91]. Therefore, continuous efforts have been ongoing to explore the optimized dose to maximize taxane adjuvant potential [92,93]. At submicromolar concentrations, PTX induces DC maturation, increases the production of proinflammatory cytokines, and up-regulates DC antigen presentation [92,93]. In summary, taxanes exert adjuvant effects on DCs, which augment their functions, including phagocytosis, antigen presentation, proinflammatory cytokine production, and T cell instruction when drug dose is optimized (Fig. 3A). In this regard, biomaterial-based DDSs provide a promising strategy to achieve optimized taxane dosing by controlling local drug exposure, release kinetics, and immune-cell uptake. Therefore, DDS-mediated dose optimization may be particularly useful for maximizing the DC-adjuvant effects of taxanes while minimizing nonspecific cytotoxicity, providing a logical basis for the following discussion of taxane DDSs in vivo.

### Effects of taxanes on T cells

In cancer immunotherapy, CD8^+^ T cells are the central effector cells that exert cellular immune responses in an antigen-specific manner and are primed mainly by DCs with the help of CD4^+^ T cells [75,83]. T_reg_s are a subtype of CD4^+^ T cells that suppress inflammatory immune responses and are critical in shaping immunological self-tolerance [81,82]. Therefore, efficient anticancer immunotherapy requires the expansion of CD8^+^ T cells in secondary lymphoid tissues, prevention of inflammatory T cell anergy, and exhaustion at the tumor bed [75,76].

In several in vitro experiments using T_reg_s and CD8^+^ T cells isolated from mice, preferential inhibition of T_reg_ proliferation was observed following PTX treatment, whereas CD8^+^ T cell proliferation was not substantially changed [94–96]. In addition, PTX markedly reduced the ability of T_reg_s to suppress CD8^+^ T cell proliferation in the MLR assay [94], which was ascribed to decreased forkhead box P3 expression and inhibited anti-inflammatory cytokine production, including transforming growth factor-β (TGF-β) and IL-10 in vitro [95,96]. These results are correlated with the depletion of T_reg_s in vivo after treatment with PTX [94–98]. Furthermore, PTX and DTX down-regulate the expression of PD-1 on Jurkat T cells in vitro [99,100].

Despite the negligible cytotoxicity of CD8^+^ T cells [94–96], PTX inhibits the function of CD8^+^ T cells [101,102]. In contrast to the substantial CD8^+^ T cell proliferation in MLR conditions to pretreat taxane to DCs before mixing with T cells [57,85,86], MLR conditions to coculture DCs and T cells simultaneously treated with PTX led to the suppression of T cell proliferation [101]. Similarly, DTX inhibits T cell activation surface marker (CD71) expression on T cells in peripheral blood mononuclear cells in vitro [102]. Moreover, the levels of integrins that govern T cell adhesion to cancer cells were substantially reduced by PTX treatment in Jurkat T cells in vitro (Fig. 3B) [103]. As the differentiation, proliferation, and activation of T cells are highly dependent on various immune cells, cytokines, and chemokines, investigating the direct effects of taxanes on T cells in vitro and correlating the in vitro results with those of the in vivo and clinical data is difficult. For instance, although the antigen-specific killing functions and cytokine secretion of CD8^+^ T cells were not changed by PTX in vitro [95,96,104], in vivo results showed PTX-mediated inhibition of the killing functions of CD8^+^ T cells [104]. However, the direct effects of CTX on other T cells have not been thoroughly investigated in vitro. Overall, taxanes have both proinflammatory and anti-inflammatory functions in T cells, depending on the type and status of T cells.

### Effects of taxanes on B cells

B cells are antibody-producing lymphocytes with APC functions that also contribute to shaping the complex immunity of the DC–T axis via cellular interactions or cytokine productions [105–107].

Studies exploring the direct in vitro effects of taxanes on the function and differentiation of B cells are limited. PTX causes RPMI-1788 B lymphoblast apoptosis by damaging DNA [108] and pretreatment with PTX prevents the adjuvant effects of lipopolysaccharide (LPS) on 70Z/3 Pre-B lymphocyte*s* in vitro [109], implying the immunosuppressive functions of PTX on B cells. However, patients treated with PTX or DTX exhibit negligible changes in B cell populations clinically [110]. In addition, an in vivo study has demonstrated that DTX acts as an adjuvant in the production of immunoglobulin G following influenza A H1N1 vaccine administration in mice [111]. Further mechanistic investigations are required to understand the roles of taxanes in the proliferation, differentiation, and activation of B cells and their functions in complex immune networks.

### Effects of taxanes on Mφs

Mφs are innate immune cells that are major phagocytes, which are classically categorized into anti-inflammatory M2 Mφs and proinflammatory M1 Mφs [78,112]. M2 Mφs support immunosuppressive environments for proliferation, extracellular matrix remodeling, angiogenesis, and tumor metastasis [78,112]. M2 Mφs in the context of cancer are also called tumor-associated Mφs (TAMs) as they are highly infiltrated in the TMEs [78,112]. In contrast, M1 Mφs elicit antibody-dependent cellular phagocytosis (ADCP) on cancer cells, damage the vascular structure around TME, and assist adaptive immune response (Fig. 3C) [78,112]. Therefore, numerous research efforts have aimed to identify drugs and DDSs to deplete TAMs and reprogram M2 Mφs into M1 Mφs for anticancer immunotherapy [113].

Taxane governs the actions, polarization, and activation status of Mφs. In murine Mφ RAW264.7, PTX exerts antimicrotubular effects by disrupting actin filaments and disintegrating the nucleus, leading to decreased cytoplasmic viscosity and cytotoxicity [114,115]. Nevertheless, PTX could potentiate the ADCP of human peripheral blood monocyte-derived Mφs on various cancer cells (cetuximab-mediated ADCP on DLD1 cells, cetuximab-mediated ADCP on SW480 cells, rituximab-mediated ADCP on Raji cells, and trastuzumab-mediated ADCP on SKBR3 cells) in vitro [116]. The enhanced ADCP effects were proportional to the PTX concentration treated on bone marrow-derived Mφs (BMDMs) but not on cancer cells [116], indicating that PTX-mediated enhanced ADCP is indirectly associated with ICD or cytotoxic effects on cancer cells [116,117]. ADCP of Mφs is directly regulated by ligation of CD47, an antiphagocytic “don’t eat me” signal, with its counterpart, signal regulatory protein α (Sirpα) expressed on Mφs [117]. Blocking the ligation with anti-CD47 or anti-Sirpα improved the ADCP ability of BMDM in vitro [117]. In this regard, PTX and CTX were identified to sensitize the ADCP ability of Mφs in conjunction with CD47–Sirpα blockades [117]. However, Sirpα, Fc**γ**RI, Fc**γ**RIIB, Fc**γ**RIII, and Fc**γ**RIV expression in Mφs remained unchanged with PTX treatment, implying the presence of alternative pathway for the enhanced taxane-induced ADCP ability [117]. Considering the enhanced ADCP ability of M1 Mφs and compromised phagocytic ability of M2 Mφs, the effects and mechanism of PTX on Mφ polarization were investigated [116]. PTX markedly stimulated M1 polarization (MHCII up-regulation and CD206 down-regulation) with the production of proinflammatory cytokines IL-1β, IL-6, and TNF-α via the nuclear factor κB (NF-κB) pathway [116,118] (Fig. 3C). In addition, the enhanced ADCP ability of M1 Mφs induced by PTX treatment is closely related to CSF1R down-regulation [116].

Taxane-mediated M1 polarization is substantially influenced by adjuvant functions of taxane on TLR4 pathway; LPS inhibitors prevent the PTX-induced TNF-α production in thioglycollate-elicited peritoneal Mφs isolated from C3H/OuJ mice [119], and pretreatment of PTX inhibits the LPS-induced expression of inducible nitric oxide synthase (iNOS) in RAW264.7 cells [120] and peritoneal Mφs [121], and LPS-induced TNF-α in peritoneal Mφs [122]. PTX-induced M1 Mφs showed enhanced IL-6, IL-12, and TNF-α production through iNOS, Krox-24, and COX-2 in a TLR4-dependent manner (Fig. 3C) [123–130].

Transcriptomic data describes the functional role of the taxane TLR4 pathway. Transcriptomics and single-cell RNA sequencing datasets of patient-derived triple-negative breast cancer (TNBC) identified Mφs as a major cell expressing TLR4 and flow cytometry dataset to validate intracellular TLR4 expression in the TME of TNBC syngeneic mouse models indicated TAMs as predominant cells expressing TLR4 in TME [131]. Additional single-cell RNA sequencing datasets suggest that the TLR4 signaling pathway was markedly up-regulated in TAMs from patients with TNBC treated with PTX, which correlate with the enhanced cross-presentation signaling pathway and antitumor efficacy [131]. The association between the TLR4 signaling pathway and cross-presentation signaling pathway in PTX treatment was investigated in vitro by analyzing the transcriptome of BMDM conditioned with IL-4 [131]. PTX treatment elevated the expression of NOX2-related genes, including CYBB and NCF2, which inhibited rapid phagolysosomal acidification, leading to enhanced antigen survival and cross-presentation (Fig. 3C) [131].

Recent in vivo findings further reveal TIM3/V-domain Ig suppressor of T cell activation (VISTA)–PTX axis [132]. In a murine model of immunoresistant cancer, a unique subset of TAMs coexpressing TIM3 and VISTA was found to dominate poorly immunogenic TMEs, exhibiting an M2-like, anti-inflammatory phenotype with an impaired type I interferon (IFN) response. Mechanistically, PTX-induced ICD in tumor cells causes the release of danger signals like HMGB1 and VISTA, which engage TAM-expressed TIM3/VISTA receptors and further suppress type I IFN signaling in these Mφs. Notably, combining PTX with TIM3/VISTA checkpoint blockade effectively repolarized the TIM3^+^VISTA^+^ TAMs to a proinflammatory (M1-like) state that directly killed cancer cells via tumor necrosis factor-related apoptosis-inducing ligand (TRAIL)-mediated apoptosis. This synergistic reprogramming of TAMs substantially blunted tumor growth and, importantly, operated through a TAM-intrinsic mechanism independent of CD8+ T cells or DCs. These results highlight not only a clinical potent of reprograming immunosuppressive Mφs into potent antitumor effectors but also a complementary strategy whereby taxane chemotherapy targeting Mφs can be paired with immune checkpoint modulation.

Various PTX-loaded DDSs have been also demonstrated to reprogram Mφs to M1 phenotypes in vitro. *nab*-PTX was taken up by M2 Mφs via macropinocytosis, resulting in the M1-like phenotypes with increased *Il1a*, *Il1b*, *Il12p40*, *Il6*, *Ifn-γ*, and decreased *Fizz1*, *Tgf-β*, and *Il-10* mRNA levels. These changes were accompanied by increased IL-12p70 and CD86 expression and decreased TGF-β and CD206 expression in vitro via the reactive oxygen species (ROS)–HMGB1–TLR4 axis [133–135]. NPs self-assembled with peptide-PTX also enabled the up-regulation of CD11c expression and down-regulation of CD206 expression in BMDMs in vitro [136]. In addition to PTX, free DTX [128], DTX-loaded NPs [137], and free CTX [138,139] led to the differentiation of M1 phenotypes in vitro, clearly indicating the conserved mechanisms of actions of taxanes in Mφs (Fig. 3C).

In summary, taxanes have a pivotal role in shaping immunostimulatory Mφs in anticancer effects; however, further studies are required to address conflicting results that PTX drives the Mφs toward immunosuppressive phenotypes with increased PD-L1 expression [140] and trigger HMGB1 release to induce peripheral neuropathy [141].

### Effects of taxanes on NK cells

**Fig. 4.**
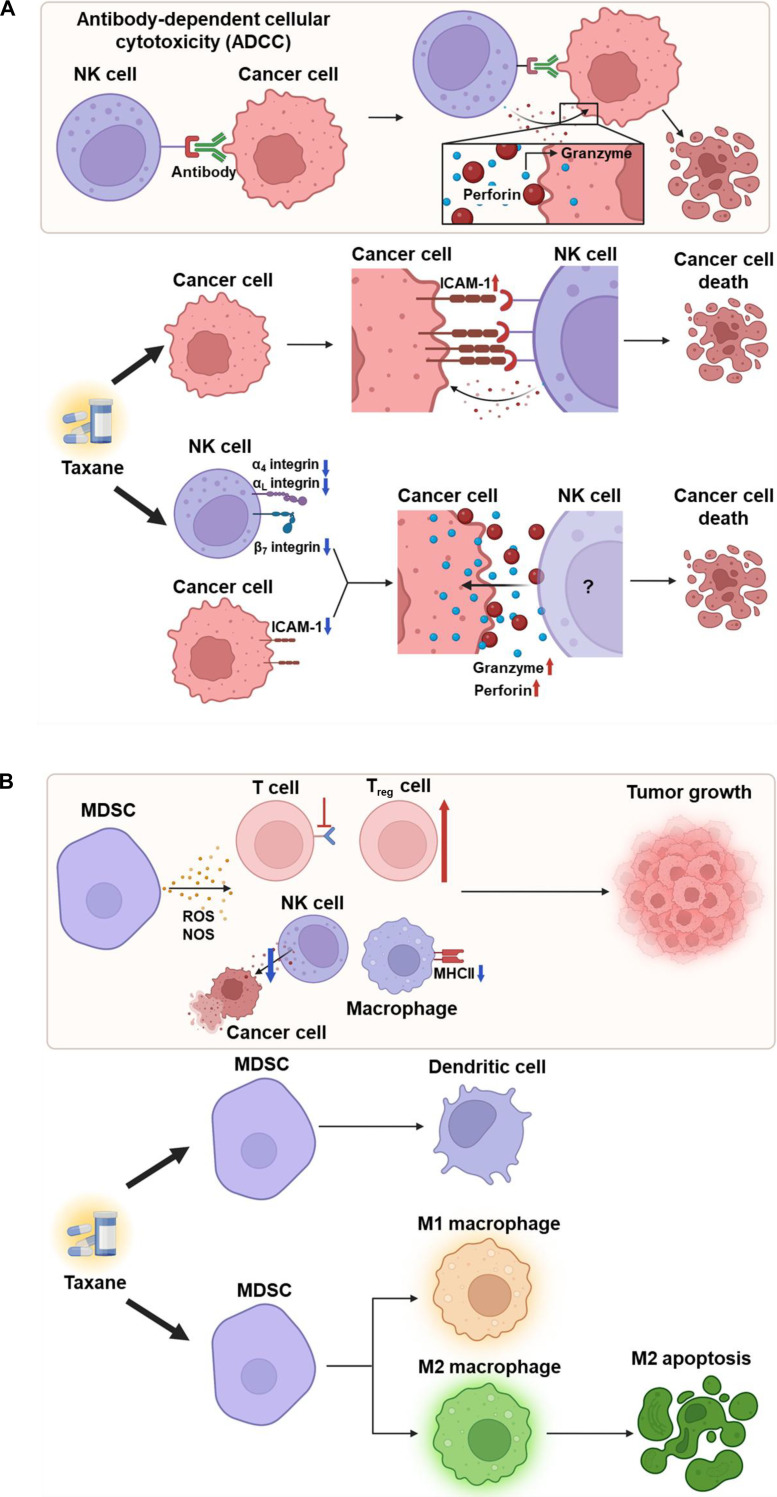
Schematic illustrations of taxane-mediated regulation of cytotoxic effector cells and immunosuppressive cells. (A) Effects of taxanes on natural killer (NK) cell interactions and the enhancement of antibody-dependent cellular cytotoxicity (ADCC) against cancer cells. ICAM-1, intercellular adhesion molecule-1. (B) Impact on immunosuppressive myeloid-derived suppressor cells (MDSCs), focusing on the inhibition of their activity and the restoration of robust antitumor immune responses. All illustrations were created using BioRender.com.

NK cells are professional killer cells among innate immune cells, which not only elicit antibody-dependent cellular cytotoxicity to recognize Fc receptors of antibodies bound to target cells and express pore-forming proteins called perforin and proteases called granzymes but also foster immunostimulatory environments to orchestrate adaptive immunity (Fig. 4A) [142]. Considering their high therapeutic potential and off-the-shelf treatment regime, chimeric antigen receptor-engineered NK cells have been extensively explored in clinical trials [143]. In addition, various anticancer drugs have been evaluated to potentiate NK cell-mediated anticancer immunotherapy, including directly activating NK cells and making cancer cells accessible for NK cell-mediated anticancer immunotherapy by inducing ligand expression for NK cell activation and recognition in cancer cells [144].

Taxanes also contribute to the sensitization of various cancer cells to NK cell-mediated cytotoxicity. Treatment of ovarian cancer cells (SKOV-3) with noncytotoxic concentrations of PTX resulted in surface intercellular adhesion molecule-1 overexpression associated with its interaction with NK cells, potentiating the NK cell-mediated lysis of cancer cells in in vitro MLR assay (Fig. 4A) [145]. Similarly, DTX stimulated NKG2D ligand expression in breast carcinoma cells (BT474 and MDA-MB-361) [146]. When DTX-pretreated human leukemia lymphoblasts (K-562) were cocultured with NK cells or peripheral blood mononuclear cells containing NK cells, NKG2D and DNAM-1 receptor expression on NK cells was markedly up-regulated but failed to promote the differentiation and proliferation of carboxyfluorescein succinimidyl ester-labeled NK cells in vitro [147]. The inhibitory effects of DTX on the susceptibility of cancer cells to NK cell-mediated cytotoxicity were also reported in an MLR assay coculturing NK cells and DTX-pretreated castration-resistant prostate cancer cells (C4-2 and CWR22Rv1), which was ascribed to the DTX-mediated up-regulation of lectin-like transcript 1 expression in CRPC cells, making them less vulnerable to NK cell cytotoxic functions [148].

Beyond what is known with respect to the conflicting effects of NK cells on cancer cells in terms of sensitization, the direct effects of taxanes on the differentiation of NK cells have been poorly investigated in vitro. Instead, the effects of taxanes on the ability of NK cells to bind to target cells and on perforin production have been investigated [149,150]. PTX treatment decreased α_4_, α_L_, and β_7_ integrin on NK cells (YT cells) and decreased intercellular adhesion molecule-1 expression on human leukemia lymphoblast cells (K562), impairing binding of NK cells to target cancer cells [149]. Nevertheless, PTX treatment in coculture system containing NK and cancer cells (BT-474 or K562) potentiated the cytotoxic functions of NK cells by elevating perforin production via a NF-κB pathway (Fig. 4A) [150]. This apparent discrepancy may reflect the context-dependent regulation of distinct steps in NK cell-mediated cytotoxicity, in which PTX can reduce adhesion-dependent NK-target cell binding whereas enhancing downstream effector activity through NF-κB-mediated perforin production in coculture conditions.

Briefly, PTX possesses contradictory antitumor effects in terms of the binding ability and cytotoxic functions of NK cells and susceptibility of cancer cells to NK cells. Accordingly, further investigations using various experimental conditions and other taxanes, including DTX and CTX, are required to elucidate the direct effects of these taxanes on NK cells.

### Effects of taxanes on MDSCs

MDSCs are immature myeloid cells that produce ROS and nitrogen species to disrupt T cell receptors, deplete amino acids required for T cell activation and proliferation, promote T_reg_ expansion, inhibit cytotoxic functions of NK, and decrease antigen presentations on Mφs, creating immunosuppressive environments (Fig. 4B) [151]. Accordingly, developing strategies to suppress MDSC recruitment to the TME, deplete MDSCs, and block their functions is warranted for effective anticancer immunotherapy [80,152].

Taxanes contribute to efficient anticancer therapies by reversing the immunosuppressive effects of MDSCs. Although low concentrations of PTX (1 and 50 nM) induce subtle apoptosis, they stimulate the differentiation of MDSCs into DCs with the ability to induce allogeneic T cell proliferation in vitro [153]. In addition, DTX (1.1 nM) induced the differentiation of MDSCs into M1 Mφs, while a partial differentiation into M2 Mφs was observed with the apoptosis in vitro (Fig. 4B) [154]. These findings are consistent with animal studies and clinical results observed when taxanes were administered at low doses [154–157]. Briefly, taxanes exert additional antitumor effects by reducing MDSCs through their differentiation into immunostimulatory cells, although further studies are needed to investigate the effects on MDSC function, their recruitment into the TME, and their subsequent impact on T_reg_s.

## Recent Advances in Taxane DDSs to Orchestrate Antitumor Immunity In Vivo

**Fig. 5.**
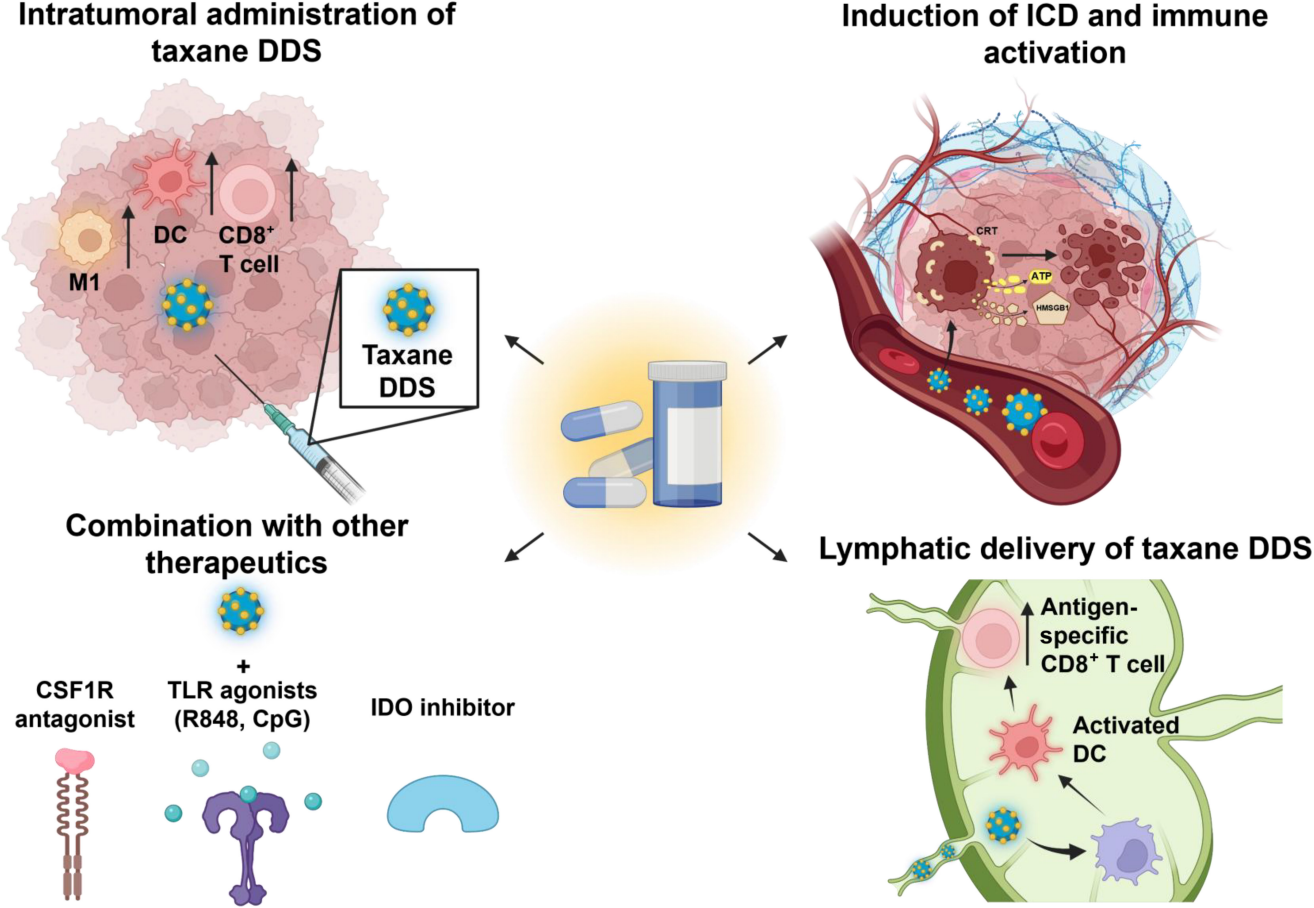
Schematic illustration of the locoregional functions of taxane drug delivery system (DDS) in anticancer immunotherapy. The illustration was created using BioRender.com.

Anticancer immunity progresses through a sequential immunological cascade comprising antigen uptake associated with ICD, DC activation, and T cell expansion. However, these processes occur in distinct anatomical locations. ICD is initiated within the TME, where dying tumor cells release CRT, ATP, and HMGB1. DCs subsequently engulf tumor antigens at tumor sites and migrate to the dLNs. Within the dLNs, DCs mature and present tumor-derived antigens to naïve T cells, orchestrating their activation and expansion into cytotoxic T lymphocytes (CTLs). These effector T cells then return to the tumor and execute targeted immune responses against residual malignant cells. This spatially distinct sequence underscores the necessity of tumor- as well as lymphatic-targeted delivery strategies to optimize antigen uptake and presentation and adaptive immune priming, ultimately enhancing the efficacy of immunotherapies [33]. Accordingly, various taxane DDSs will be discussed in the following text (Table 1), focusing on each locational aspect of antitumor immune response (Fig. 5).

**Table 1.** Recent advances in taxane DDSs

Therapeutic methodology	Drugs (cotreated)	Delivery injection	DDS type	Immune responses in vitro	Immune responses in vivo	Ref.
Intratumoral administration	PTX	Intratumoral	Polyethyleneimine–lithocholic acid conjugate NPs	Induces DC maturation and promotes TNF-α and IL-1β production	Maintains relatively low levels of MDSCs and T_reg_s by silencing siPD-L1	[158]
				Exposes CRT on CT26 and B16F10	Induces CD8^+^ T cells	
Intratumoral administration	PTX (NO) (aCTLA-4)	Intratumoral (intraperitoneal)	PTX-loaded NPs formulated by multivalent host–guest interactions	Induces synergistic cytotoxicity	Exerts substantial systemic DC expansion and activation	[40]
				ICD effects are similar to those of free PTX	Impairs instruction and expansion of CD8^+^ T cells	
				Induces higher DC activation than that of free PTX		
Intratumoral administration	DTX (anti-mCTLA-4)		Submicron particle loading DTX	-	Thoracic metastasis substantially reduced after cotreatment of anti-mCTLA-4	[159]
					Efficient antitumor immunotherapeutic effects	
					Markedly increases CD8^+^ T cells in tumor bed and systemic blood stream	
Intratumoral administration	DTX (aPD-L1)	Intratumoral	DTX-loaded nanoprecipitation		Enhances antitumor effects by reversing immunosuppressive TME	[160]
Intratumoral administration	DTX (CpG)	Intratumoral	DTX-loaded nanodisc	Dose-dependent cellular nanodisc uptake	Markedly increases median survival with no systemic toxicity	[161]
					Elicits antitumor immunological memory after treatment of DTX-loaded nanodisc-CpG	
Intratumoral administration	DTX	Intraperitoneal (local implantation)	Electrospun nanofibrous mat	-	Suppresses M2-like TAMs and elevates the M1/M2 Mφ ratio, enhancing CD8^+^ T cell infiltration in tumor tissues	[163]
EPR effect	PTX (STING)	Intravenous	Integrin αXXXβ1 targeting PTX NP	Exerts higher CRT, ATP, and HMGB1 induction than that of free PTX	Efficiently uptake into 4T1 tumor	[48]
					Substantial expansion of CD8^+^ T cells and reduction of T_reg_s after cotreatment with STING agonists	
EPR effect	DTX (aPD-1)	Intravenous	DTX-loaded NP	Induces CRT, ATP, and HMGB1 on 4T1 cells	Blocks tumor growth and inhibits tumor metastasis	[59]
					Triggers efficient CD8^+^ and CD4^+^ T cells after cotreatment with aPD-1	
EPR effect	PTX (x-ray)	Intravenous	Nanoassembly composed of PTX, human serum albumin, and aPD-1	High ICD factor (CRT, HMGB1, and ATP) levels in the following combination with x-ray	Promotes CD8^+^ T cell infiltration and its IFN-γ-expressing subtype	[58]
				Markedly enhances DCs maturation after combination with x-ray	Eliminates M2 Mφ, T_reg_s, and MDSCs in tumor tissues following combination with x-ray	
EPR effect	PTX (aPD-1)	Intravenous	pH-responsive and MMP-sensitive charge-conversion micelle loading PTX	Produces the highest apoptotic tumor cell levels	Up-regulates PD-L1 and CRT levels	[41]
					Substantially increases tumor filtration of CD8^+^ T cells after cotreatment with aPD-1	
					Exhibits the highest proinflammatory cytokines (IFN-γ and TNF-α) levels after treatment with aPD-1	
EPR effect	PTX (aPD-L1)	Intravenous	PEG-PLA NP loading PTX	Increases T cells, DC infiltration and activation, and TAMs	Induces ICD-inducing agent (CRT, ERp57, ATP, and HMGB1)	[51]
					Markedly increases T cells and DC infiltration and function	
				Decreases T_reg_s and MDSCs	Codelivery of aPD-1 leads to higher therapeutic effects than that of single aPD-1 or NP therapy	
EPR effect	PTX (aPD-L1)	Intravenous	NP composed of 3-(2-nitrophenyl) propionic acid-PTX	CRT and HMGB1 expression on MDA-MB-231 cells	Increases recruitment and infiltration of CD8^+^ T cells in tumor tissues when codelivered with aPD-L1	[43]
				Promotes DC maturation and enhances T cell antigen expression		
EPR effect	PTX (aPD-L1)	Intravenous	BTB permeable peptide modified redox-responsive micelle	Induces CRT, ATP, and HMGB1 expression on GL261 cells	Substantially enhances the expansion of activated DCs, M1 Mφ, CD8^+^ CTLs, and CD8^+^ T/T_reg_ ratio	[46]
				Elicits sufficient ICD, allowing DCs and Mφs to activate antigen presenting function	Reduces MDSCs levels in tumor bed	
EPR effect	PTX	Intravenous	Self-assembled NP + R848 liposomes	-	Augments intratumoral CD8^+^ T cell infiltration (~3.2-fold) and depletes T_reg_s (~73.7% reduction); also promotes DC maturation and elevates proinflammatory cytokines (TNF-α, IFN-γ)	[165]
EPR effect	PTX	Intravenous	Hybrid micelle composed of PEG-PCL and PCL-PEI/siRNA	Enhances IFN-γ secretion on T cells and CRT exposure on tumor cells	Suppresses tumor growth via CD8^+^ T cell expansion	[45]
				Increases CD8^+^ T and CD4^+^ T cell numbers	Enhances CD8^+^ CTLs/T_reg_s ratios and CD4^+^ T_eff_s/T_reg_s ratios	
EPR effect	PTX	Intravenous	PEI-PLGA NP coloading PTX and CRISPRT/Cas9-Cdk5 plasmid	Substantially high transfection efficacy in B16F10 cells	Reduces PD-L1 expression in CT26 colorectal tumor	[39]
				Efficiently elicits ICD effects in weakly acidic environment	Leads to CD8^+^ T cell expansion, M1 Mφ, and reduced T_reg_ in tumor cells	
Synergize the therapeutic mechanism of taxane DDS	PTX	Intravenous	Nanoassembly composed of dimerized PTX and IDO inhibitor	High TNF-α, IFN-γ, and IL-6 levels secretion by taxane- and DDS-treated DC	Exerts antitumor immunotherapeutic effects by synergizing taxane and IDO inhibitor	[166]
				Induces IDO expression in cancer cells		
Synergize the therapeutic mechanism of taxane DDS	PTX	Intravenous	Red blood cell membrane-camouflaged NP loaded with PTX and IDO inhibitors	IFN-γ released by taxane- and DDS-treated DC	Increases the amount of CD8^+^ T cells and M1 Mφs by synergizing taxane with IDO inhibitor	[167]
				Induces IDO expression in cancer cells		
Synergize the therapeutic mechanism of taxane DDS	PTX	Intravenous	Albumin-bound PTX–IDO inhibitor conjugate	IFN-γ released by taxane- and DDS-treated DC	Increases tumor accumulation with the systemic administration by synergizing taxane with IDO inhibitor	[168]
				Induces the expression of IDO in cancer cells	High amount of active mDCs, CD4^+^ T, and CD8^+^ T cells	
Synergize the therapeutic mechanism of taxane DDS	DTX	Intravenous	Micelle chemically conjugated with DTX and IDO inhibitor	Efficient cellular uptake and cancer cell selectivity	Motivates the intratumoral secretion of IFN-γ after taxane DDS treatment	[169]
					Suppresses IDO expression compared with that of free DTX	
Synergize the therapeutic mechanism of taxane DDS	DTX	Intravenous	pH-degradable PVA nanogel containing DTX and IDO inhibitor	Efficient IDO activity inhibition	Elevation of NK cells and the survival and activity of CD8^+^ T cells	[170]
				Substantially activates T cells proliferation by strong IDO inhibitor	Reduces the number of MDSCs by synergizing taxane with IDO inhibitor	
Synergize the therapeutic mechanism of taxane DDS	DTX	Intravenous	Micellar DTX together with R848 micelle	Facilitates DC maturation and M1 Mφ polarization	Induces Mφ production of proinflammatory phenotypes following sequential administration	[137]
Synergize the therapeutic mechanism of taxane DDS	PTX (Fru + R848)	Intravenous	Self-assembled NP + R848 liposomes	Substantially promotes BMDC maturation (↑CD80/CD86); cotreatment with R848 further heightens DC activation and drives M2→M1 Mφ repolarization	Elicits robust immunity, markedly expanding intratumoral CD8^+^ T cells and the M1/M2 Mφ ratio while inducing high levels of IL-12 and TNF-α; achieves complete tumor rejection in ~37.5% of mice with long-term immune memory	[171]
Synergize the therapeutic mechanism of taxane DDS	PTX	Intravenous	Integrin-targeting micellar gemcitabine and PTX combined with CpG	Enhances DC maturation associated codelivery of CpG adjuvant and PTX	Substantially induces DC recruitment and activation, and TNF-α and IFN-γ production	[172]
Synergize the therapeutic mechanism of taxane DDS	PTX + PCSK9 inhibitor (PF-06446846)	Intravenous	HA/R8-RGD dual-targeted albumin NPs	Induces ICD (CRT, ATP, and HMGB1); up-regulates tumor MHCI; promotes DC maturation and cytokine secretion (IFN-γ and TNF-α)	Increases CD8^+^ T cells (CD69^+^CD25^+^); enhances DCs in tumor-draining lymph nodes; induces complete tumor rejection and long-term immunity	[174]
Synergize the therapeutic mechanism of taxane DDS	PTX	Intratumoral	Encodes adenovirus incorporated into anionic lipid containing PTX	Increases the level of IL-12 compared with that produced by free encoding and anionic lipid + encoding genes	Produces high levels of IL-12 and IFN-γ	[176]
Synergize the therapeutic mechanism of taxane DDS	PTX (celecoxib [COX-2i])	Intravenous	cRGD-modified liposomal NP	Promotes DC maturation; increases IL-12 and TNF-α; decreases IL-10 and TGF-β	Enhances infiltration of CD8^+^ and CD4^+^ T cells; promotes memory CD8^+^ cells; reduces MDSCs and T_reg_s; promotes M1 and suppresses M2 TAMs	[177]
Synergize the therapeutic mechanism of taxane DDS	CTX	Intravenous	Nanoassembly constructed from STAT3 inhibitor and CTX	Increases the levels of DNA fragments released by apoptotic cells following B16F10 cells treatment	Suppresses cancer cell immunosuppressive IL-10 and VEGF secretion	[178]
Lymphatic delivery	PTX	Ipsilateral	PTX-loaded poly(propylene sulfide) NP	-	DCs were expanded and activated by TLR activation within NPs	[34]
					Antigen-specific CD8^+^ T cells levels were increased in TdLNs	
					Marginal antitumor effects on B16F10 tumor-bearing mice	
Lymphatic delivery	PTX (aPD-1)	Intravenous (intraperitoneal)	PTX-loaded poly(propylene sulfide) NP	-	Induces systemic antitumor effects on primary and secondary tumors	[187]
		Intratumoral (intratumoral)			Ipsilateral administration substantially reduces tumor volume that is associated with high NP accumulation	
		Ipsilateral (intratumoral)			Ipsilateral aPD-1 administration was more effective than that of intraperitoneal and is comparable to that if intratumoral	
		Ipsilateral (ipsilateral)			Exhibits antitumor therapeutic effects and CD8^+^ T cell responses comparable to those observed with the intratumoral administration of PTX-loaded NPs and aPD-1	
Lymphatic delivery	PTX (aPD-1) (aPD-L1)	Intratumoral	Nanosuspension containing PTX, R848, and IDO inhibitor	Leads to apoptosis with increased ICD markers (CRT and HMGB1) on 4T1 cells	Primed T cells in the lymph node filtrate to indicate possible systemic immunity in tumors at distal locations	[42]
				Induces proinflammatory cytokines (IFN-γ, TNF- α, and IL-10) in BMDC		

DDS, drug delivery system; PTX, paclitaxel; DTX, docetaxel; CTX, cabazitaxel; ATP, adenosine triphosphate; HMGB1; high-mobility group protein box 1; CRT, calreticulin; MMP, matrix metalloproteinase; DC, dendritic cell; Mφ, macrophage; NK, natural killer; T_reg_s, regulatory T cells; MDSC, myeloid-derived suppressor cell; BMDC, bone-marrow dendritic cell; MHCI, major histocompatibility complex I; TNF-α, tumor necrosis factor-α; TLR, Toll-like receptor; TGF-β, transforming growth factor-β; TAM, tumor-associated Mφ; IFN-γ, interferon-γ; CTL, cytotoxic T lymphocyte; EPR, enhanced permeation and retention; STING, stimulator of interferon gene; PEG, polyethylene glycol; PLA, poly(lactic acid); BTB, blood–tumor barrier; PEI, polyethyleneime; siRNA, small interfering RNA; PLGA, poly(lactic-co-glycolic) acid; IDO, indoleamine-2,3-dioxygenase; STAT3, signal transducer and activator of transcription 3; TdLNs, tumor-draining lymph nodes; TME, tumor microenvironment; PVA, poly(vinyl alcohol); NP, nanoparticle; T_eff_s, effector T cells; VEGF, vascular endothelial growth factor

First, the intratumoral administration of taxane DDSs directly initiates antitumor immunity in the TME in vivo [40,50,57,89,158–161]. For instance, polyethyleneimine–lithocholic acid conjugate NPs loaded with PTX showed substantial anticancer effects on CT26 cancer cells in vitro, while inducing negligible cytotoxicity in BMDCs and splenocytes. Its intratumoral administration showed the increased populations of DCs, MDSCs, T_reg_s, and CD8^+^ T cells, and M1/M2 Mφs ratio. Our group reported PTX-loaded NPs formulated by multivalent host–guest interactions as an antitumor immunotherapeutic to induce ICD and activate DCs independently [40]. Although the ICD effects of PTX-loaded NPs were similar to those of free PTX in vitro, the NPs induced higher activation (CD86^+^ and MHCII) of DCs than free PTX in vitro [40]. Their intratumoral administration resulted in a markedly higher therapeutic index in vivo when codelivered with nitric oxide, an important signaling molecule [162], and aCTLA-4, an ICB in vivo [40]. In addition to PTX DDSs, intratumoral delivery of submicron particle-loaded DTX [159], DTX-loaded nanoprecipitates codelivered with PD-L1 inhibitors [160], and DTX-loaded nanodiscs with CpG adjuvant [161] showed efficient antitumor immunotherapeutic effects, with substantially increased CD8^+^ T cells in the tumor bed as well as systemic bloodstream. Beyond NP systems, an implantable DTX-loaded electrospun nanofibrous mat was reported to reshape the tumor immune microenvironment at the surgical margin. This localized DTX DDS depleted CD206^+^ M2-like Mφs in the tumor and correspondingly increased F4/80^+^ iNOS^+^ M1-like Mφs, raising the overall M1/M2 TAM ratio. In parallel, intratumoral CD8^+^ cytotoxic T cell infiltration was markedly enhanced by the sustained DTX release. These findings show that local sustained-release taxane delivery can reprogram TAM immunophenotypes (fewer M2-like TAMs, more M1-like TAMs) and boost antitumor CD8^+^ T cell presence in the TME [163].

Second, various taxane DDSs that enable the efficient accumulation of taxane in the tumor bed via enhanced permeation and retention effects and active targeting ability have been developed, providing detailed insights into antitumor immune processes. An integrin α_5_β_1_ targeting PTX NPs exerted higher CRT, ATP, and HMGB1 induction than free PTX in vitro, efficiently uptake into 4T1 tumor via enhanced permeation and retention effects and active targeting in vivo, and its combination therapy with a stimulator of interferon gene agonist adjuvant led to efficient antitumor effects with substantial expansion of CD8^+^ T cells and reduced T_reg_s [48]. DTX-loaded NPs [59] and nanoassemblies composed of CTX, human serum albumin, and aPD-1 [58] also showed CRT, ATP, and HMGB1 induction on 4T1 cells in vitro, followed by efficient CD8^+^ T cell, M1 Mφs responses, and antitumor therapy in vivo. As discussed above, cancer cells threatened by taxanes can up-regulate the expression of immune checkpoints and ligands to evade immune surveillance [41,45,57,58,63,67,68,70,71]. Indeed, pH-responsive and matrix metalloproteinase-sensitive charge-conversion micelles loaded with PTX induced CRT expression and up-regulated PD-L1 [41]. In addition, CT26 cells were induced to express CRT and ATP HMGB1 and up-regulate PD-L1 by a polyethylene glycol (PEG)–poly(lactic acid) (PLA) NPs, followed by phagocytosis by BMDCs in vitro [51]. The immunosuppressive functions of taxane-induced PD-L1 is inhibited by the codelivery of aPD-L1 [43,46,51,164]. PEG-PLA NPs loaded with PTX led to a substantial expansion of activated (MHCII and CD86^+^) DCs (CD11c) and CD8^+^ T cells along with reduced T_reg_s and MDSCs (CD11b^+^Gr-1^+^) in the tumor in vivo [51]. However, codelivery of aPD-1 with PEG-PLA NPs loaded with PTX led to greater therapeutic effects than aPD-1 or NP therapy [51]. Likewise, a NP composed of 3-(2-nitrophenyl) propionic acid-PTX showed CRT and HMGB1 expression in MDA-MB-231 and 4T1 cells in vitro, engendering substantial antitumor effects with an increased number of CD8^+^ T cells in tumor tissues when combined with aPD-L1 in vivo compared to that of the saline treatment group [43]. Blood–tumor barrier permeable peptide modified redox-responsive micelles loading PTX, and aPD-L1 also induce CRT, ATP, and HMGB1 expression on GL261 cells in vitro, efficiently absorb into tumor brain tumor in vivo, and markedly enhance the expansion of activated (MHCII, CD80^+^, and CD86^+^) DCs, M1(CD86^+^) Mφs with M1/M2 (CD206^+^) Mφs ratio, IFN-γ^+^, and granzyme B^+^ CD8^+^ T cells with CD8^+^ T/T_reg_ ratio and reduce the MDSCs in the tumor bed in vivo, which resulted in their markedly improved survival in vivo [46]. Similarly, the PD-1/PD-L1 axis was exploited by cloaking PTX dimer loading NPs with PD-1-rich cell membranes (PD-1@PTX_2_ NPs) [165]. These biomimetic NPs selectively bind PD-L1 on tumor cells, acting as a decoy to block the PD-1/PD-L1 axis while delivering PTX, thereby augmenting T cell activity in the TME. In 4T1 breast tumor models, PD-1@PTX_2_ NPs improved intratumoral drug accumulation and induced robust antitumor immunity, evidenced by a 3.2-fold increase in CD8^+^ T cell infiltration and ~74% reduction in intratumoral T_reg_s. This was accompanied by enhanced DC maturation (CD80^+^/CD86^+^) in tumors, collectively leading to markedly inhibited tumor growth and prolonged survival compared to free PTX (intravenous). In addition to antagonistic antibodies, the immunosuppressive functions of taxane DDS-induced PD-L1 is regulated by inhibiting its expression using gene silencing and editing technologies. A hybrid micelle composed of PEG-polycaprolactone (PCL) and PCL-polyethyleneime (PEI) loading PTX and small interfering RNA specific to PD-L1 mRNA into the PCL core and PEI shell, respectively, resulted in tumor growth suppression with CD8^+^ T cell expansion and PD-L1 silencing in B16F10 tumor in vivo [45]. Similarly, a PEI-poly(lactic-co-glycolic) acid NP coloading PTX and CRISPRT/Cas9-Cdk5 plasmid reduced the expression of PD-L1 in CT26 colorectal tumor, leading to the CD8^+^ T cell expansion and M1 Mφs with reduced T_reg_s in tumor [39]. Notably, this work demonstrated PTX-mediated ICD in tumor tissue in vivo [39], in contrast to other reports showing ICD effects only in vitro [40,41,43,45,46,48,50,51,57–59,63,67,68,70,71,90,158–161,164].

Third, other therapeutic methodologies could be used to synergize the therapeutic mechanisms of taxane DDSs in the tumor bed. Indoleamine-2,3-dioxygenase (IDO) is an enzyme involved in tumor cell evasion from immune surveillance, tumor growth and metastasis, impairment of CD8^+^ T cell functions, CD8^+^ T cell apoptosis, and increase in T_reg_s [166–170]. IFN-γ is released by taxane- or taxane DDSs-treated DCs, which induces IDO expression in cancer cells to foster immunosuppressive environment [166,167]. Accordingly, a nanoassembly comprising dimerized PTX and IDO inhibitor [166], red blood cell membrane-camouflaged NPs loaded with PTX and IDO inhibitor [167], albumin-bound PTX–IDO inhibitor conjugates [168], a micelle chemically conjugated with DTX and IDO inhibitor [169], and pH-degradable poly(vinyl alcohol) nanogels containing DTX and IDO inhibitor [170] were developed, which exploited the cytotoxic and ICD functions of taxane as well as exerted efficient antitumor immunotherapeutic effects by synergizing the inhibition of the immunosuppressive IDO pathway [166–170]. In addition, TLR7/8a agonist (R848) to facilitate DC maturation and M1 Mφ polarization [137,171], TLR9 agonistic nucleotides (CpG) to act as an adjuvant for DCs, Mφs, and NK cells [172], CSF1R antagonist to block TAM recruitment [173], PCSK9 inhibitor to induce tumor ICD and enhance antigen presentation [174], immunostimulatory cytokines [175], cytokine-encoding plasmid DNA [176], COX-2 inhibitor to suppress synthesis of prostaglandin E_2_ attenuating the immunogenicity of dying tumor cells [177], or signal transducer and activator of transcription 3 inhibitor to suppress immunosuppressive IL-10 and vascular endothelial growth factor secretion from cancer cells [178], were also coformulated or codelivered with taxane DDS to explore the synergistic antitumor immunotherapy. Furthermore, photothermal therapy, which converts electromagnetic light radiation to heat to destroy tumor cells [179–181], and photodynamic therapy, which uses light-activated photosensitizers to generate ROS to kill cancer cells [52,181], were exploited together with taxane DDSs to improve ICD effects in the taxane-mediated antitumor immunotherapy process. Emerging evidence reveals that beyond drug selection, ratiometric dose optimization is pivotal in cancer immunotherapy. For instance, a nanomedicine coloading PTX and rapamycin at a precise 5:1 ratio remodeled the immunosuppressive TME and suppressed lung metastasis in a triple-negative breast cancer model far more effectively than other dose ratios [182].

Fourth, the lymphatic delivery [183–185] of taxane DDS is under exploration, as it mainly exploits the adjuvant effects of taxanes for antitumor immunotherapy, excluding cytotoxic and ICD functions. Thomas’s group was the first to investigate the adjuvant effects of PTX in vivo [34]. Efficient lymphatic delivery requires rational control of nanocarrier physicochemical properties. In particular, hydrodynamic size is one of the most critical parameters governing interstitial transport and lymphatic drainage; nanocarriers in the 10- to 50-nm range, and in some contexts up to approximately 100 nm, generally exhibit favorable lymph node accumulation after locoregional administration, whereas larger particles tend to remain entrapped at the injection site. Surface electrostatics also influence transport through the extracellular matrix, with negatively charged or charge-shielded surfaces often facilitating interstitial movement by reducing nonspecific interactions. In addition, appropriate hydrophilic surface modification, such as PEGylation, can improve colloidal stability and reduce nonspecific binding, thereby supporting lymphatic trafficking and subsequent interactions with APCs in dLNs [184]. PTX-loaded poly(propylene sulfide) (PPS) NPs with an approximately 30-nm diameter and a Pluronic-stabilized surface were injected into the forelimb skin ipsilateral to the tumor and selectively drained into tumor-draining lymph nodes (TdLNs) [34]. Despite negligible accumulation in tumor [186], DCs were expanded and activated, and antigen-specific CD8^+^ T cells were increased in TdLNs, leading to marginal antitumor effects in B16F10 tumor-bearing mice [34]. As PPS NP administration into the forelimb skin contralateral to the tumor could not provoke an immunostimulatory response or antitumor effects, these results demonstrate the immune axis between TdLNs and tumors in taxane-mediated antitumor therapy. Thomas’s group compared the biodistribution, therapeutic efficacy, and immune response of intravenous and ipsilateral administration of PTX-loaded PPS NPs [187]. Notably, ipsilateral administration elicited significantly higher antitumor effects in terms of tumor volume in E0771 orthotopic tumor models, which correlated with a higher accumulation of NPs in inguinal dLNs that are directly linked with orthotopic E0771 tumor [187]. In line with previous findings demonstrating that ipsilateral administration of ICBs was more effective than systemic intraperitoneal administration and comparable to that of intratumoral administration [186], ipsilateral administration of PTX-loaded PPS with aPD-1 therapy induced systemic antitumor effects on both primary and secondary tumors and enabled dose reduction compared to that of intravenous administration [187]. In particular, ipsilateral administration of PTX-loaded NPs combined with intratumoral administration of aPD-1 exhibited antitumor therapeutic effects and CD8^+^ T cell responses comparable to those observed with intratumoral administration of PTX-loaded NPs and aPD-1 [187]. This suggests that the adjuvant effects of PTX play a major role in antitumor mechanisms in vivo. Regardless of whether a taxane is delivered to the tumor through intratumoral administration or systemic administration with targeted DDSs, its effects extend beyond cytotoxic activity and ICD induction, which inevitably triggers lymphatic drainage, thereby facilitating the adjuvant effects of taxane**s** in parallel. Jin et al. reported a nanosuspension containing PTX, R848, and an IDO inhibitor as a locoregional intratumoral injectable depot for systemic antitumor immunotherapy [42]. As expected, PTX in the nanosuspension not only led to apoptosis with increased ICD markers (CRT and HMGB1) on 4T1 cells in vitro but also induced the proinflammatory cytokines (IFN-γ, TNF-α, and IL-10) in BMDCs in vitro [42]. As no experimental comparisons were discovered using a nanosuspension containing only or lacking PTX [42], the specific impact of intratumoral PTX delivery on immunity in TdLNs could not be clearly determined. Nevertheless, as the nanosuspension was efficiently delivered to the TdLNs [42], we can infer that the adjuvant effects of PTX also contribute to the overall therapeutic efficacy of the nanosuspension. Additionally, although direct experimental evidence has not been provided, it was speculated that the adjuvant effects of taxanes in lymph nodes contributes to the therapeutic efficacy of taxane DDSs that have been delivered to the tumor tissue [39–46,48,50–52,57–59,70,71,89,136,137,158–162,164,166–170,172,173,175,176,178–181].

## Conclusion

Taxane-based chemotherapeutics have been used extensively for decades because of their potent cytotoxic effects against a broad spectrum of cancers. However, their role in modulating the immune system has only recently been recognized. This review highlights the emerging paradigm in which taxane-mediated immunomodulation contributes markedly to antitumor efficacy beyond the classical mechanisms of microtubule stabilization and apoptosis induction.

A key insight from recent findings is the spatial segregation of taxane-induced immune processes, wherein ICD occurs within the TME, antigen uptake by DCs is followed by their migration to dLNs, and T cell priming and expansion predominantly occurs in secondary lymphoid organs. Considering the compartmentalized nature of antitumor immunity, conventional systemic taxane administration may not fully exploit the immunomodulatory potential. This has driven the development of DDSs that enable precise tumor- and lymphatic-targeted delivery, thereby optimizing both direct cytotoxic effects and immune stimulation.

Despite the promising preclinical and clinical outcomes, several challenges remain in fully harnessing the immunostimulatory effects of taxanes. First, although various taxane DDSs have demonstrated superior accumulation in tumors and lymphoid tissues, the ideal formulation for maximizing ICD and immune priming remains unclear. Studies have suggested that NP-based DDSs enhance ICD induction and antigen presentation; however, the optimal size, surface charge, and composition for balancing tumor retention and lymphatic trafficking require further exploration. Additionally, the type and composition of NPs substantially influence their biodistribution, cellular uptake, and immunostimulatory potential, necessitating careful therapeutically based selection [188–190]. Second, considering that TdLNs serve as critical sites for antigen processing and T cell activation, lymphatic-targeted taxane DDSs present a novel strategy to potentiate immune responses. However, further investigations are needed to refine administration routes and dosing strategies to maximize therapeutic benefits while minimizing systemic toxicity. Third, recent studies have highlighted the countertherapeutic effects of taxanes, wherein both PTX and DTX can promote lymphangiogenesis [191,192]. Accordingly, careful consideration of lymphatic-targeted taxane DDSs is essential, as unintentional lymphangiogenesis could counteract the therapeutic benefits and promote metastatic progression. Fourth, the synergistic potential of taxane-based DDSs with ICB therapy and other immunomodulatory drugs has been highlighted in preclinical studies. Taxane-induced PD-L1 up-regulation in tumor cells provides a strong rationale for combining taxane DDSs with aPD-1/PD-L1 therapy to enhance T cell activation and overcome immune evasion. Furthermore, coformulation or coadministration with adjuvants, cytokines, or TLR agonists could further amplify the immune-boosting effects of taxanes, warranting systematic evaluation in preclinical and clinical settings. Fifth, although taxane-mediated immune activation is well documented, its long-term effects on the mechanism behind immune memory and tumor recurrence remain unclear. Future studies should investigate whether taxane-induced immune responses generate durable antitumor immunity or lead to immune exhaustion and resistance.

To translate these advances into clinical practice, rationally designed taxane DDSs should be evaluated in controlled clinical trials. Recent clinical progress in tumor-activated T cell engager platforms further supports the importance of localized immune modulation in modern oncology. For example, PSMA-targeted tumor-activated T cell engagers, including JANX007 and JANX014, are being clinically evaluated in metastatic castration-resistant prostate cancer, including taxane-naïve patient populations and combination settings with androgen receptor pathway inhibition. Although these approaches are not taxane DDSs themselves, they illustrate the rapid clinical expansion of tumor-localized immune activation strategies and reinforce the rationale for developing taxane-based DDSs that can integrate cytotoxic chemotherapy with spatially controlled immunomodulation. Moreover, personalized taxane DDS-based immunotherapy strategies tailored to individual tumor immune profiles can maximize clinical outcomes while minimizing off-target effects. Recent advances in patient-derived multiomics analyses and artificial intelligence/machine learning-assisted data interpretation may further refine taxane-based DDS strategies. By analyzing tumor specimens at genomic, transcriptomic, proteomic, and immune-microenvironmental levels, these platforms could support patient stratification, prediction of taxane responsiveness, and customized optimization of DDS composition, targeting, combination therapy, and release kinetics for more precise next-generation cancer treatment [193].

In conclusion, taxane-mediated immunomodulation represents a paradigm shift in cancer therapy, transforming taxanes from traditional cytotoxic agents into potent immunomodulators. By leveraging innovative DDSs and combinatorial strategies, taxane-based chemo-immunotherapy has the potential to redefine next-generation cancer treatments.

## Data Availability

No datasets were generated or analyzed during the current study.

## References

[1] Ojima I, Lichtenthal B, Lee S, Wang C, Wang X. Taxane anticancer agents: A patent perspective. Expert Opin Ther Pat. 2016;26(1):1–20. 26651178 10.1517/13543776.2016.1111872 PMC4941984

[2] Herbst RS, Khuri FR. Mode of action of docetaxel - A basis for combination with novel anticancer agents. Cancer Treat Rev. 2003;29(5):407–415. 12972359 10.1016/s0305-7372(03)00097-5

[3] Sweeney CJ, Miller KD, Sissons SE, Nozaki S, Heilman DK, Shen J, George W, Sledge GW Jr. The antiangiogenic property of docetaxel is synergistic with a recombinant humanized monoclonal antibody against vascular endothelial growth factor or 2-methoxyestradiol but antagonized by endothelial growth factors. Cancer Res. 2001;61(8):3369–3372. 11309294

[4] Vacca A, Ribatti D, Iurlaro M, Merchionne F, Nico B, Ria R, Dammacco F. Docetaxel versus paclitaxel for antiangiogenesis. J Hematother Stem Cell Res. 2002;11(1):103–118. 11847007 10.1089/152581602753448577

[5] Galletti E, Magnani M, Renzulli ML, Botta M. Paclitaxel and docetaxel resistance: Molecular mechanisms and development of new generation taxanes. ChemMedChem. 2007;2(7):920–942. 17530726 10.1002/cmdc.200600308

[6] Holohan C, Van Schaeybroeck S, Longley DB, Johnston PG. Cancer drug resistance: An evolving paradigm. Nat Rev Cancer. 2013;13(10):714–726. 24060863 10.1038/nrc3599

[7] Januchowski R, Sterzynska K, Zaorska K, Sosinska P, Klejewski A, Brazert M, Nowicki M, Zabel M. Analysis of MDR genes expression and cross-resistance in eight drug resistant ovarian cancer cell lines. J Ovarian Res. 2016;9(1):65. 27756418 10.1186/s13048-016-0278-z PMC5069986

[8] Martinelli C, Biglietti M. Nanotechnological approaches for counteracting multidrug resistance in cancer. Cancer Drug Resist. 2020;3(4):1003–1020. 35582219 10.20517/cdr.2020.47 PMC8992571

[9] Huang Y, Cole SP, Cai T, Cai YU. Applications of nanoparticle drug delivery systems for the reversal of multidrug resistance in cancer. Oncol Lett. 2016;12(1):11–15. 27347092 10.3892/ol.2016.4596 PMC4907090

[10] Van Zuylen L, Verweij J, Sparreboom A. Role of formulation vehicles in taxane pharmacology. Invest New Drugs. 2001;19(2):125–141. 11392447 10.1023/a:1010618632738

[11] Rajappa S, Joshi A, Doval DC, Batra U, Rajendranath R, Deo A, Biswas G, Bajpai P, Tilak TVS, Kane S, et al. Novel formulations of docetaxel, paclitaxel and doxorubicin in the management of metastatic breast cancer. Oncol Lett. 2018;16(3):3757–3769. 30127986 10.3892/ol.2018.9057 PMC6096158

[12] Roy A, Bhattacharyya M, Ernsting MJ, May JP, Li SD. Recent progress in the development of polysaccharide conjugates of docetaxel and paclitaxel. Wiley Interdiscip Rev Nanomed Nanobiotechnol. 2014;6(4):349–368. 24652678 10.1002/wnan.1264 PMC4057951

[13] Louage B, De Wever O, Hennink WE, De Geest BG. Developments and future clinical outlook of taxane nanomedicines. J Control Release. 2017;253:137–152. 28336374 10.1016/j.jconrel.2017.03.027

[14] Hanif MF, Ahmad R, Khalid K, Tabassum M, Akram F. Targeted delivery of nanoparticle based taxanes for breast cancer treatment: A review. Trends Drug Deliv. 2020;6:36–51.

[15] Sumera, Anwar A, Ovais M, Khan A, Raza A. Docetaxel-loaded solid lipid nanoparticles: A novel drug delivery system. IET Nanobiotechnol. 2017;11(6):621–629.

[16] Zhao P, Astruc D. Docetaxel nanotechnology in anticancer therapy. ChemMedChem. 2012;7(6):952–972. 22517723 10.1002/cmdc.201200052

[17] Zhang L, Zhang N. How nanotechnology can enhance docetaxel therapy. Int J Nanomedicine. 2013;8:2927–2941. 23950643 10.2147/IJN.S46921 PMC3742154

[18] Dwivedi P, Han S, Mangrio F, Fan R, Dwivedi M, Zhu Z, Huang F, Wu Q, Khatik R, Cohn DE, et al. Engineered multifunctional biodegradable hybrid microparticles for paclitaxel delivery in cancer therapy. Mater Sci Eng C Mater Biol Appl. 2019;102:113–123. 31146981 10.1016/j.msec.2019.03.009

[19] Cho JK, Hong JM, Han T, Yang HK, Song SC. Injectable and biodegradable poly(organophosphazene) hydrogel as a delivery system of docetaxel for cancer treatment. J Drug Target. 2013;21(6):564–573. 23594096 10.3109/1061186X.2013.776055

[20] Voci S, Gagliardi A, Molinaro R, Fresta M, Cosco D. Recent advances of taxol-loaded biocompatible nanocarriers embedded in natural polymer-based hydrogels. Gels. 2021;7(2):33. 33804970 10.3390/gels7020033 PMC8103278

[21] Bhatnagar S, Bankar NG, Kulkarni MV, Venuganti VVK. Dissolvable microneedle patch containing doxorubicin and docetaxel is effective in 4T1 xenografted breast cancer mouse model. Int J Pharm. 2019;556:263–275. 30557681 10.1016/j.ijpharm.2018.12.022

[22] Vishnu P, Roy V. Safety and efficacy of nab-paclitaxel in the treatment of patients with breast cancer. Breast Cancer. 2011;5:53–65. 21603258 10.4137/BCBCR.S5857 PMC3091407

[23] Yardley DA. Nab-paclitaxel mechanisms of action and delivery. J Control Release. 2013;170(3):365–372. 23770008 10.1016/j.jconrel.2013.05.041

[24] Al-Hajeili M, Azmi AS, Choi M. Nab-paclitaxel: Potential for the treatment of advanced pancreatic cancer. Onco Targets Ther. 2014;7:187–192. 24523592 10.2147/OTT.S40705 PMC3921002

[25] Miele E, Spinelli GP, Miele E, Tomao F, Tomao S. Albumin-bound formulation of paclitaxel (Abraxane® ABI-007) in the treatment of breast cancer. Int J Nanomedicine. 2009;4:99–105. 19516888 10.2147/ijn.s3061 PMC2720743

[26] Gradishar WJ, Krasnojon D, Cheporov S, Makhson AN, Manikhas GM, Clawson A, Bhar P. Significantly longer progression-free survival with nab-paclitaxel compared with docetaxel as first-line therapy for metastatic breast cancer. J Clin Oncol. 2009;27(22):3611–3619. 19470941 10.1200/JCO.2008.18.5397

[27] Zhu L, Chen L. Progress in research on paclitaxel and tumor immunotherapy. Cell Mol Biol Lett. 2019;24(1):40. 31223315 10.1186/s11658-019-0164-y PMC6567594

[28] Jones SE, Erban J, Overmoyer B, Budd GT, Hutchins L, Lower E, Laufman L, Sundaram S, Urba WJ, Pritchard KI, et al. Randomized phase III study of docetaxel compared with paclitaxel in metastatic breast cancer. J Clin Oncol. 2005;23(24):5542–5551. 16110015 10.1200/JCO.2005.02.027

[29] Fossella FV. Docetaxel in second-line treatment of non-small-cell lung cancer. Clin Lung Cancer. 2002;3 Suppl 2:S23–S28. 14720344 10.3816/clc.2002.s.010

[30] Waldman AD, Fritz JM, Lenardo MJ. A guide to cancer immunotherapy: From T cell basic science to clinical practice. Nat Rev Immunol. 2020;20(11):651–668. 32433532 10.1038/s41577-020-0306-5 PMC7238960

[31] Francis DM, Thomas SN. Progress and opportunities for enhancing the delivery and efficacy of checkpoint inhibitors for cancer immunotherapy. Adv Drug Deliv Rev. 2017;114:33–42. 28455187 10.1016/j.addr.2017.04.011 PMC5581991

[32] Grosser R, Cherkassky L, Chintala N, Adusumilli PS. Combination immunotherapy with CAR T cells and checkpoint blockade for the treatment of solid tumors. Cancer Cell. 2019;36(5):471–482. 31715131 10.1016/j.ccell.2019.09.006 PMC7171534

[33] Kim J, Manspeaker MP, Thomas SN. Augmenting the synergies of chemotherapy and immunotherapy through drug delivery. Acta Biomater. 2019;88:1–14. 30769136 10.1016/j.actbio.2019.02.012

[34] Thomas SN, Vokali E, Lund AW, Hubbell JA, Swartz MA. Targeting the tumor-draining lymph node with adjuvanted nanoparticles reshapes the anti-tumor immune response. Biomaterials. 2014;35(2):814–824. 24144906 10.1016/j.biomaterials.2013.10.003

[35] Javeed A, Ashraf M, Riaz A, Ghafoor A, Afzal S, Mukhtar MM. Paclitaxel and immune system. Eur J Pharm Sci. 2009;38(4):283–290. 19733657 10.1016/j.ejps.2009.08.009

[36] Mahanty S, Prigent A, Garraud O. Immunogenicity of infectious pathogens and vaccine antigens. BMC Immunol. 2015;16(1):31. 26021448 10.1186/s12865-015-0095-y PMC4446803

[37] Krysko DV, Garg AD, Kaczmarek A, Krysko O, Agostinis P, Vandenabeele P. Immunogenic cell death and DAMPs in cancer therapy. Nat Rev Cancer. 2012;12(12):860–875. 23151605 10.1038/nrc3380

[38] Kepp O, Senovilla L, Kroemer G. Immunogenic cell death inducers as anticancer agents. Oncotarget. 2014;5(14):5190–5191. 25114034 10.18632/oncotarget.2266 PMC4170601

[39] Tu K, Deng H, Kong L, Wang Y, Yang T, Hu Q, Hu M, Yang C, Zhang Z. Reshaping tumor immune microenvironment through acidity-responsive nanoparticles featured with CRISPR/Cas9-mediated programmed death-ligand 1 attenuation and chemotherapeutics-induced immunogenic cell death. ACS Appl Mater Interfaces. 2020;12(14):16018–16030. 32192326 10.1021/acsami.9b23084

[40] Kim J, Sestito LF, Im S, Kim WJ, Thomas SN. Poly(cyclodextrin)-polydrug nanocomplexes as synthetic oncolytic virus for locoregional melanoma chemoimmunotherapy. Adv Funct Mater. 2020;30(16): Article 1908788. 33071710 10.1002/adfm.201908788 PMC7566879

[41] Su Z, Xiao Z, Wang Y, Huang J, An Y, Wang X, Shuai X. Codelivery of anti-PD-1 antibody and paclitaxel with matrix metalloproteinase and pH dual-sensitive micelles for enhanced tumor chemoimmunotherapy. Small. 2020;16(7): Article e1906832. 31990457 10.1002/smll.201906832

[42] Jin SM, Lee SN, Kim JE, Yoo YJ, Song C, Shin HS, Phuengkham H, Lee CH, Um SH, Lim YT. Overcoming chemoimmunotherapy-induced immunosuppression by assemblable and depot forming immune modulating nanosuspension. Adv Sci. 2021;8(19): Article e2102043. 10.1002/advs.202102043 PMC8498862 34363349

[43] Duan X-C, Peng L-Y, Yao X, Xu M-Q, Li H, Zhang S-Q, Li Z-Y, Wang J-R, Feng Z-H, Wang G-X, et al. The synergistic antitumor activity of 3-(2-nitrophenyl) propionic acid-paclitaxel nanoparticles (NPPA-PTX NPs) and anti-PD-L1 antibody inducing immunogenic cell death. Drug Deliv. 2021;28(1):800–813. 33866918 10.1080/10717544.2021.1909180 PMC8079060

[44] Qin T, Xu X, Zhang Z, Li J, You X, Guo H, Sun H, Liu M, Dai Z, Zhu H. Paclitaxel/sunitinib-loaded micelles promote an antitumor response in vitro through synergistic immunogenic cell death for triple-negative breast cancer. Nanotechnology. 2020;31(36): Article 365101. 32434167 10.1088/1361-6528/ab94dc

[45] Tang X, Rao J, Yin S, Wei J, Xia C, Li M, Mei L, Zhang Z, He Q. PD-L1 knockdown via hybrid micelle promotes paclitaxel induced cancer-immunity cycle for melanoma treatment. Eur J Pharm Sci. 2019;127:161–174. 30366077 10.1016/j.ejps.2018.10.021

[46] Zhang Z, Xu X, Du J, Chen X, Xue Y, Zhang J, Yang X, Chen X, Xie J, Ju S. Redox-responsive polymer micelles co-encapsulating immune checkpoint inhibitors and chemotherapeutic agents for glioblastoma therapy. Nat Commun. 2024;15(1):1118. 38320994 10.1038/s41467-024-44963-3 PMC10847518

[47] Gu Z, Da Silva CG, Hao Y, Schomann T, Camps MGM, van der Maaden K, Liu Q, Ossendorp F, Cruz LJ. Effective combination of liposome-targeted chemotherapy and PD-L1 blockade of murine colon cancer. J Control Release. 2023;353:490–506. 36460179 10.1016/j.jconrel.2022.11.049

[48] Qiu X, Qu Y, Guo B, Zheng H, Meng F, Zhong Z. Micellar paclitaxel boosts ICD and chemo-immunotherapy of metastatic triple negative breast cancer. J Control Release. 2022;341:498–510. 34883139 10.1016/j.jconrel.2021.12.002

[49] Lau TS, Chan LKY, Man GCW, Wong CH, Lee JHS, Yim SF, Cheung TH, McNeish IA, Kwong J. Paclitaxel induces immunogenic cell death in ovarian cancer via TLR4/IKK2/SNARE-dependent exocytosis. Cancer Immunol Res. 2020;8(8):1099–1111. 32354736 10.1158/2326-6066.CIR-19-0616

[50] Kwon S, Meng F, Tamam H, Gadalla HH, Wang J, Dong B, Hopf Jannasch AS, Ratliff TL, Yeo Y. Systemic delivery of paclitaxel by find-me nanoparticles activates antitumor immunity and eliminates tumors. ACS Nano. 2024;18(4):3681–3698. 38227965 10.1021/acsnano.3c11445 PMC11025439

[51] Yang Q, Shi G, Chen X, Lin Y, Cheng L, Jiang Q, Yan X, Jiang M, Li Y, Zhang H, et al. Nanomicelle protects the immune activation effects of paclitaxel and sensitizes tumors to anti-PD-1 immunotherapy. Theranostics. 2020;10(18):8382–8399. 32724476 10.7150/thno.45391 PMC7381738

[52] Feng B, Niu Z, Hou B, Zhou L, Li Y, Yu H. Enhancing triple negative breast cancer immunotherapy by ICG-templated self-assembly of paclitaxel nanoparticles. Adv Funct Mater. 2019;30(6): Article 1906605.

[53] Flieswasser T, Van Loenhout J, Freire Boullosa L, Van den Eynde A, De Waele J, Van Audenaerde J, Lardon F, Smits E, Pauwels P, Jacobs J. Clinically relevant chemotherapeutics have the ability to induce immunogenic cell death in non-small cell lung cancer. Cells. 2020;9(6):1474. 32560232 10.3390/cells9061474 PMC7349161

[54] Wang W, Qin S, Zhao L. Docetaxel enhances CD3+ CD56+ cytokine-induced killer cells-mediated killing through inducing tumor cells phenotype modulation. Biomed Pharmacother. 2015;69:18–23. 25661332 10.1016/j.biopha.2014.10.026

[55] Hodge JW, Garnett CT, Farsaci B, Palena C, Tsang KY, Ferrone S, Gameiro SR. Chemotherapy-induced immunogenic modulation of tumor cells enhances killing by cytotoxic T lymphocytes and is distinct from immunogenic cell death. Int J Cancer. 2013;133(3):624–636. 23364915 10.1002/ijc.28070 PMC3663913

[56] Haruna M, Hirata M, Iwahori K, Kanazawa T, Yamamoto Y, Goto K, Kawashima A, Morimoto-Okazawa A, Funaki S, Shintani Y, et al. Docetaxel upregulates HMGB1 levels in non-small cell lung cancer. Biol Pharm Bull. 2020;43(3):399–403. 32115500 10.1248/bpb.b19-00702

[57] Choi B, Jung H, Yu B, Choi H, Lee J, Kim DH. Sequential MR image-guided local immune checkpoint blockade cancer immunotherapy using ferumoxytol capped ultralarge pore mesoporous silica carriers after standard chemotherapy. Small. 2019;15(52): Article e1904378. 31697036 10.1002/smll.201904378 PMC7027959

[58] Wang J, Li J, Wu Y, Xu X, Qian X, Lei Y, Liu H, Zhang Z, Li Y. ROS-responsive nanocomplex of aPD-L1 and cabazitaxel improves intratumor delivery and potentiates radiation-mediated antitumor immunity. Nano Lett. 2022;22(20):8312–8320. 36226914 10.1021/acs.nanolett.2c03227

[59] Wang Y, Wang Q, Wang X, Yao P, Dai Q, Qi X, Yang M, Zhang X, Huang R, Yang J, et al. Docetaxel-loaded pH/ROS dual-responsive nanoparticles with self-supplied ROS for inhibiting metastasis and enhancing immunotherapy of breast cancer. J Nanobiotechnology. 2023;21(1):286. 37608285 10.1186/s12951-023-02013-y PMC10464340

[60] Wykes MN, Lewin SR. Immune checkpoint blockade in infectious diseases. Nat Rev Immunol. 2018;18(2):91–104. 28990586 10.1038/nri.2017.112 PMC5991909

[61] Tu L, Guan R, Yang H, Zhou Y, Hong W, Ma L, Zhao G, Yu M. Assessment of the expression of the immune checkpoint molecules PD-1, CTLA4, TIM-3 and LAG-3 across different cancers in relation to treatment response, tumor-infiltrating immune cells and survival. Int J Cancer. 2020;147(2):423–439. 31721169 10.1002/ijc.32785

[62] Blanc-Durand F, Genestie C, Galende EY, Gouy S, Morice P, Pautier P, Maulard A, Mesnage S, Le Formal A, Brizais C, et al. Distribution of novel immune-checkpoint targets in ovarian cancer tumor microenvironment: A dynamic landscape. Gynecol Oncol. 2021;160(1):279–284. 33162175 10.1016/j.ygyno.2020.09.045

[63] Yoon HK, Kim TH, Park S, Jung H, Quan X, Park SJ, Han J, Lee A. Effect of anthracycline and taxane on the expression of programmed cell death ligand-1 and galectin-9 in triple-negative breast cancer. Pathol Res Pract. 2018;214(10):1626–1631. 30139555 10.1016/j.prp.2018.08.009

[64] Fang J, Chen F, Liu D, Gu F, Chen Z, Wang Y. Prognostic value of immune checkpoint molecules in breast cancer. Biosci Rep. 2020;40(7): Article BSR20201054. 32602545 10.1042/BSR20201054 PMC7340863

[65] Hu F-F, Liu C-J, Liu L-L, Zhang Q, Guo A-Y. Expression profile of immune checkpoint genes and their roles in predicting immunotherapy response. Brief Bioinform. 2021;22(3): Article bbaa176. 32814346 10.1093/bib/bbaa176

[66] Dai C, Geng R, Wang C, Wong A, Qing M, Hu J, Sun Y, Lo AW, Li J. Concordance of immune checkpoints within tumor immune contexture and their prognostic significance in gastric cancer. Mol Oncol. 2016;10(10):1551–1558. 27720576 10.1016/j.molonc.2016.09.004 PMC5423138

[67] Zhao Y, Wang Z, Shi X, Liu T, Yu W, Ren X, Zhao H. Effect of chemotherapeutics on in vitro immune checkpoint expression in non-small cell lung cancer. Technol Cancer Res Treat. 2023;22: Article 15330338231202307. 37728201 10.1177/15330338231202307 PMC10515539

[68] Majidi M, Safaee S, Amini M, Baghbanzadeh A, Hajiasgharzadeh K, Hashemzadeh S, Sandoghchian Shotorbani S, Mokhtarzadeh A, Baradaran B. The effects of chemotherapeutic drugs on PD-L1 gene expression in breast cancer cell lines. Med Oncol. 2021;38(12):147. 34687372 10.1007/s12032-021-01556-0

[69] Nasiri H, Ahmadpour Youshanlui M, Valedkarimi Z, Ahmadian Heris J, Mokhtarzadeh A, Shanehbandi D, Ahmadi H, Jafarizadeh A, Baradaran B. Impact of chemotherapeutic agents on PD-L1, CTLA-4, and VISTA gene expression in cervical cancer cell lines: An in vitro study. Indian J Gynecol Oncol. 2024;22(2):65.

[70] Hu D, Zhang W, Xiang J, Li D, Chen Y, Yuan P, Shao S, Zhou Z, Shen Y, Tang J. A ROS-responsive synergistic delivery system for combined immunotherapy and chemotherapy. Mater Today Bio. 2022;14: Article 100284. 10.1016/j.mtbio.2022.100284 PMC9130108 35647515

[71] Wang Y, Yu J, Li D, Zhao L, Sun B, Wang J, Wang Z, Zhou S, Wang M, Yang Y, et al. Paclitaxel derivative-based liposomal nanoplatform for potentiated chemo-immunotherapy. J Control Release. 2022;341:812–827. 34953979 10.1016/j.jconrel.2021.12.023

[72] Li Y, Ji Y, Shen L, Yin X, Huang T, Deng B, Guo H, Wu Y, Chen Y. Clinical efficacy of combination therapy of an immune checkpoint inhibitor with taxane plus platinum versus an immune checkpoint inhibitor with fluorouracil plus platinum in the first-line treatment of patients with locally advanced, metastatic, or recurrent esophageal squamous cell carcinoma. Front Oncol. 2022;12: Article 1015302. 36605427 10.3389/fonc.2022.1015302 PMC9808083

[73] Soliman HH. Nab-paclitaxel as a potential partner with checkpoint inhibitors in solid tumors. Onco Targets Ther. 2017;10:101–112. 28053544 10.2147/OTT.S122974 PMC5189972

[74] Fizazi K, Mella PG, Castellano D, Minatta JN, Kalebasty AR, Shaffer D, Limón JCV, López HMS, Armstrong AJ, Horvath L, et al. Nivolumab plus docetaxel in patients with chemotherapy-naive metastatic castration-resistant prostate cancer: Results from the phase II CheckMate 9KD trial. Eur J Cancer. 2022;160:61–71. 34802864 10.1016/j.ejca.2021.09.043

[75] Mellman I, Coukos G, Dranoff G. Cancer immunotherapy comes of age. Nature. 2011;480(7378):480–489. 22193102 10.1038/nature10673 PMC3967235

[76] Sterner RC, Sterner RM. CAR-T cell therapy: Current limitations and potential strategies. Blood Cancer J. 2021;11(4):69. 33824268 10.1038/s41408-021-00459-7 PMC8024391

[77] Wang W, Liu Y, He Z, Li L, Liu S, Jiang M, Zhao B, Deng M, Wang W, Mi X, et al. Breakthrough of solid tumor treatment: CAR-NK immunotherapy. Cell Death Discov. 2024;10(1):40. 38245520 10.1038/s41420-024-01815-9 PMC10799930

[78] Mantovani A, Allavena P, Marchesi F, Garlanda C. Macrophages as tools and targets in cancer therapy. Nat Rev Drug Discov. 2022;21(11):799–820. 35974096 10.1038/s41573-022-00520-5 PMC9380983

[79] Wu Y, Yi M, Niu M, Mei Q, Wu K. Myeloid-derived suppressor cells: An emerging target for anticancer immunotherapy. Mol Cancer. 2022;21(1):184. 36163047 10.1186/s12943-022-01657-y PMC9513992

[80] Dai X, Ren L, Liu M, Cai H, Zhang H, Gong Q, Gu Z, Luo K. Nanomedicines modulating myeloid-derived suppressor cells for improving cancer immunotherapy. Nano Today. 2021;39: Article 101163.

[81] Tanaka A, Sakaguchi S. Regulatory T cells in cancer immunotherapy. Cell Res. 2017;27(1):109–118. 27995907 10.1038/cr.2016.151 PMC5223231

[82] Tay C, Tanaka A, Sakaguchi S. Tumor-infiltrating regulatory T cells as targets of cancer immunotherapy. Cancer Cell. 2023;41(3):450–465. 36917950 10.1016/j.ccell.2023.02.014

[83] Galluzzi L, Senovilla L, Zitvogel L, Kroemer G. The secret ally: Immunostimulation by anticancer drugs. Nat Rev Drug Discov. 2012;11(3):215–233. 22301798 10.1038/nrd3626

[84] Alloatti A, Kotsias F, Magalhaes JG, Amigorena S. Dendritic cell maturation and cross-presentation: Timing matters! Immunol Rev. 2016;272(1):97–108. 27319345 10.1111/imr.12432 PMC6680313

[85] Tanaka H, Matsushima H, Mizumoto N, Takashima A. Classification of chemotherapeutic agents based on their differential in vitro effects on dendritic cells. Cancer Res. 2009;69(17):6978–6986. 19706756 10.1158/0008-5472.CAN-09-1101 PMC2769260

[86] Nakashima H, Tasaki A, Kubo M, Kuroki H, Matsumoto K, Tanaka M, Nakamura M, Morisaki T, Katano M. Effects of docetaxel on antigen presentation-related functions of human monocyte-derived dendritic cells. Cancer Chemother Pharmacol. 2005;55(5):479–487. 15726369 10.1007/s00280-004-0918-7

[87] Pfannenstiel LW, Lam SS, Emens LA, Jaffee EM, Armstrong TD. Paclitaxel enhances early dendritic cell maturation and function through TLR4 signaling in mice. Cell Immunol. 2010;263(1):79–87. 20346445 10.1016/j.cellimm.2010.03.001 PMC2862830

[88] Marin-Esteban V, Charron D, Gelin C, Mooney N. Chemotherapeutic agents targeting the tubulin cytoskeleton modify LPS-induced cytokine secretion by dendritic cells and increase antigen presentation. J Immunother. 2010;33(4):364–370. 20386470 10.1097/CJI.0b013e3181cd1094

[89] Seth A, Heo MB, Lim YT. Poly (γ-glutamic acid) based combination of water-insoluble paclitaxel and TLR7 agonist for chemo-immunotherapy. Biomaterials. 2014;35(27):7992–8001. 24954733 10.1016/j.biomaterials.2014.05.076

[90] Joo H-G. Altered maturation of dendritic cells by taxol, an anticancer drug. J Vet Sci. 2003;4(3):229–234. 14685027

[91] John J, Ismail M, Riley C, Askham J, Morgan R, Melcher A, Pandha H. Differential effects of paclitaxel on dendritic cell function. BMC Immunol. 2010;11(1):14. 20302610 10.1186/1471-2172-11-14 PMC2850888

[92] Shurin GV, Tourkova IL, Kaneno R, Shurin MR. Chemotherapeutic agents in noncytotoxic concentrations increase antigen presentation by dendritic cells via an IL-12-dependent mechanism. J Immunol. 2009;183(1):137–144. 19535620 10.4049/jimmunol.0900734 PMC4005417

[93] Kaneno R, Shurin GV, Tourkova IL, Shurin MR. Chemomodulation of human dendritic cell function by antineoplastic agents in low noncytotoxic concentrations. J Transl Med. 2009;7(1):58. 19591684 10.1186/1479-5876-7-58 PMC2716306

[94] Zhang L, Dermawan K, Jin M, Liu R, Zheng H, Xu L, Zhang Y, Cai Y, Chu Y, Xiong S. Differential impairment of regulatory T cells rather than effector T cells by paclitaxel-based chemotherapy. Clin Immunol. 2008;129(2):219–229. 18771959 10.1016/j.clim.2008.07.013

[95] Liu N, Zheng Y, Zhu Y, Xiong S, Chu Y. Selective impairment of CD4+CD25+Foxp3+ regulatory T cells by paclitaxel is explained by Bcl-2/Bax mediated apoptosis. Int Immunopharmacol. 2011;11(2):212–219. 21115120 10.1016/j.intimp.2010.11.021

[96] Zhu Y, Liu N, Xiong SD, Zheng YJ, Chu YW. CD4^+^Foxp3^+^ regulatory T-cell impairment by paclitaxel is independent of toll-like receptor 4. Scand J Immunol. 2011;73(4):301–308. 21223350 10.1111/j.1365-3083.2011.02514.x

[97] Vicari AP, Luu R, Zhang N, Patel S, Makinen SR, Hanson DC, Weeratna RD, Krieg AM. Paclitaxel reduces regulatory T cell numbers and inhibitory function and enhances the anti-tumor effects of the TLR9 agonist PF-3512676 in the mouse. Cancer Immunol Immunother. 2009;58(4):615–628. 18802696 10.1007/s00262-008-0586-2 PMC11030133

[98] Sevko A, Kremer V, Falk C, Umansky L, Shurin MR, Shurin GV, Umansky V. Application of paclitaxel in low non-cytotoxic doses supports vaccination with melanoma antigens in normal mice. J Immunotoxicol. 2012;9(3):275–281. 22449053 10.3109/1547691X.2012.655343

[99] Franchini D-M, Lanvin O, Tosolini M, Patras de Campaigno E, Cammas A, Péricart S, Scarlata CM, Lebras M, Rossi C, Ligat L, et al. Microtubule-driven stress granule dynamics regulate inhibitory immune checkpoint expression in T cells. Cell Rep. 2019;26(1):94–107 e7. 30605689 10.1016/j.celrep.2018.12.014

[100] Zhang C, Li F, Li J, Xu Y, Wang L, Zhang Y. Docetaxel down-regulates PD-1 expression via STAT3 in T lymphocytes. Clin Lung Cancer. 2018;19(5):e675–e683. 29844001 10.1016/j.cllc.2018.04.010

[101] Cao L, Sun D, Cruz T, Moscarello MA, Ludwin SK, Whitaker JN. Inhibition of experimental allergic encephalomyelitis in the Lewis rat by paclitaxel. J Neuroimmunol. 2000;108(1–2):103–111. 10900343 10.1016/s0165-5728(00)00268-x

[102] Si M-S, Imagawa DK, Ji P, Wei X, Holm B, Kwok J, Lee M, Reitz BA, Borie DC. Immunomodulatory effects of docetaxel on human lymphocytes. Invest New Drugs. 2003;21(3):281–290. 14578678 10.1023/a:1025408425660

[103] Bhan V, Mader JS, Hoskin DW. In vitro exposure to paclitaxel modulates integrin expression by human T lymphocytes and inhibits T cell adhesion to breast carcinoma cells. Oncol Rep. 2004;11(4):893–897. 15010891

[104] Robinson MJ, Ronchese F, Miller JH, La Flamme AC. Paclitaxel inhibits killing by murine cytotoxic T lymphocytes in vivo but not in vitro. Immunol Cell Biol. 2010;88(3):291–296. 19997079 10.1038/icb.2009.96

[105] Wennhold K, Shimabukuro-Vornhagen A, von Bergwelt-Baildon M. B cell-based cancer immunotherapy. Transfus Med Hemother. 2019;46(1):36–46. 31244580 10.1159/000496166 PMC6558332

[106] Sarvaria A, Madrigal JA, Saudemont A. B cell regulation in cancer and anti-tumor immunity. Cell Mol Immunol. 2017;14(8):662–674. 28626234 10.1038/cmi.2017.35 PMC5549607

[107] Wang J-Z, Zhang Y-H, Guo X-H, Zhang H-Y, Zhang Y. The double-edge role of B cells in mediating antitumor T-cell immunity: Pharmacological strategies for cancer immunotherapy. Int Immunopharmacol. 2016;36:73–85. 27111515 10.1016/j.intimp.2016.04.018

[108] Amato SF, Swart JM, Berg M, Wanebo HJ, Mehta SR, Chiles TC. Transient stimulation of the c-Jun-NH_2_-terminal kinase/activator protein 1 pathway and inhibition of extracellular signal-regulated kinase are early effects in paclitaxel-mediated apoptosis in human B lymphoblasts. Cancer Res. 1998;58(2):241–247. 9443400

[109] Lee M, Jeon YJ. Paclitaxel-induced immune suppression is associated with NF-κB activation via conventional PKC isotypes in lipopolysaccharide-stimulated 70Z/3 pre-B lymphocyte tumor cells. Mol Pharmacol. 2001;59(2):248–253. 11160860 10.1124/mol.59.2.248

[110] Tong AW, Seamour B, Lawson JM, Ordonez G, Vukelja S, Hyman W, Richards D, Stein L, Maples PB, Nemunaitis J. Cellular immune profile of patients with advanced cancer before and after taxane treatment. Am J Clin Oncol. 2000;23(5):463–472. 11039505 10.1097/00000421-200010000-00007

[111] Chen J, Yuan L, Fan Q, Su F, Chen Y, Hu S. Adjuvant effect of docetaxel on the immune responses to influenza A H1N1 vaccine in mice. BMC Immunol. 2012;13(1):36. 22769233 10.1186/1471-2172-13-36 PMC3447692

[112] Komohara Y, Fujiwara Y, Ohnishi K, Takeya M. Tumor-associated macrophages: Potential therapeutic targets for anti-cancer therapy. Adv Drug Deliv Rev. 2016;99(Pt B):180–185. 26621196 10.1016/j.addr.2015.11.009

[113] Xia Y, Rao L, Yao H, Wang Z, Ning P, Chen X. Engineering macrophages for cancer immunotherapy and drug delivery. Adv Mater. 2020;32(40): Article e2002054. 32856350 10.1002/adma.202002054

[114] Chen Y-C, Wu C-M. To study the effect of paclitaxel on the cytoplasmic viscosity of murine macrophage immune cell RAW 264.7 using self-developed optical tweezers system. Jpn J Appl Phys. 2012;51(12R): Article 127001.

[115] Yadav A, Mandal MK, Dubey KK. In vitro cytotoxicity study of cyclophosphamide, etoposide and paclitaxel on monocyte macrophage cell line Raw 264.7. Indian J Microbiol. 2020;60(4):511–517. 33088001 10.1007/s12088-020-00896-1 PMC7539243

[116] Cao X, Chen J, Li B, Dang J, Zhang W, Zhong X, Wang C, Raoof M, Sun Z, Yu J. Promoting antibody-dependent cellular phagocytosis for effective macrophage-based cancer immunotherapy. Sci Adv. 2022;8(11): Article eabl9171. 35302839 10.1126/sciadv.abl9171 PMC8932662

[117] Cao X, Wang Y, Zhang W, Zhong X, Gunes EG, Dang J, Wang J, Epstein AL, Querfeld C, Sun Z, et al. Targeting macrophages for enhancing CD47 blockade–elicited lymphoma clearance and overcoming tumor-induced immunosuppression. Blood. 2022;139(22):3290–3302. 35134139 10.1182/blood.2021013901 PMC9164740

[118] Perera P-Y, Qureshi N, Vogel SN. Paclitaxel (Taxol)-induced NF-κB translocation in murine macrophages. Infect Immun. 1996;64(3):878–884. 8641795 10.1128/iai.64.3.878-884.1996 PMC173851

[119] Manthey C, Qureshi N, Stütz P, Vogel S. Lipopolysaccharide antagonists block taxol-induced signaling in murine macrophages. J Exp Med. 1993;178(2):695–702. 8101863 10.1084/jem.178.2.695 PMC2191120

[120] Li MH, Kang JS, Kim H, Jeon YH. Pretreatment of macrophages with paclitaxel inhibits iNOS expression. Toxicol Res. 2006;22(2):103–107.

[121] Kirikae T, Kirikae F, Oghiso Y, Nakano M. Microtubule-disrupting agents inhibit nitric oxide production in murine peritoneal macrophages stimulated with lipopolysaccharide or paclitaxel (Taxol). Infect Immun. 1996;64(8):3379–3384. 8757879 10.1128/iai.64.8.3379-3384.1996 PMC174233

[122] Nakano M, Tominaga K, Saito S, Kirikae F, Lin S, Fumero CL, Ojima I, Kirikae T. Lipopolysaccharide-and paclitaxel (Taxol)-induced tolerance in murine peritoneal macrophages. J Endotoxin Res. 1999;5(1–2):102–106.

[123] Kim YM, Paik S-G. Induction of expression of inducible nitric oxide synthase by Taxol in murine macrophage cells. Biochem Biophys Res Commun. 2005;326(2):410–416. 15582593 10.1016/j.bbrc.2004.11.043

[124] Mullins DW, Burger CJ, Elgert KD. Paclitaxel enhances macrophage IL-12 production in tumor-bearing hosts through nitric oxide. J Immunol. 1999;162(11):6811–6818. 10352302

[125] Moos PJ, Fitzpatrick FA. Taxane-mediated gene induction is independent of microtubule stabilization: Induction of transcription regulators and enzymes that modulate inflammation and apoptosis. Proc Natl Acad Sci USA. 1998;95(7):3896–3901. 9520464 10.1073/pnas.95.7.3896 PMC19934

[126] Kirikae T, Ojima I, Fuero-Oderda C, Lin S, Kirikae F, Hashimoto M, Nakano M. Structural significance of the acyl group at the C-10 position and the A ring of the taxane core of paclitaxel for inducing nitric oxide and tumor necrosis factor production by murine macrophages. FEBS Lett. 2000;478(3):221–226. 10930572 10.1016/s0014-5793(00)01858-5

[127] Mullins DW, Martins RS, Burger CJ, Elgert KD. Tumor cell-derived TGF-β and IL-10 dysregulate paclitaxel-induced macrophage activation. J Leukoc Biol. 2001;69(1):129–137. 11200057

[128] Cassidy PB, Moos PJ, Kelly RC, Fitzpatrick FA. Cyclooxygenase-2 induction by paclitaxel, docetaxel, and taxane analogues in human monocytes and murine macrophages: Structure-activity relationships and their implications. Clin Cancer Res. 2002;8(3):846–855. 11895918

[129] Yamaguchi T, Fushida S, Yamamoto Y, Tsukada T, Kinoshita J, Oyama K, Miyashita T, Tajima H, Ninomiya I, Munesue S, et al. Low-dose paclitaxel suppresses the induction of M2 macrophages in gastric cancer. Oncol Rep. 2017;37(6):3341–3350. 28440494 10.3892/or.2017.5586

[130] Wanderley CW, Colón DF, Luiz JPM, Oliveira FF, Viacava PR, Leite CA, Pereira JA, Silva CM, Silva CR, Silva RL, et al. Paclitaxel reduces tumor growth by reprogramming tumor-associated macrophages to an M1 profile in a TLR4-dependent manner. Cancer Res. 2018;78(20):5891–5900. 30104241 10.1158/0008-5472.CAN-17-3480

[131] Vadevoo SMP, Kang Y, Gunassekaran GR, Lee S-M, Park M-S, Jo DG, Kim S-K, Lee H, Kim WJ, Lee B. IL4 receptor targeting enables nab-paclitaxel to enhance reprogramming of M2-type macrophages into M1-like phenotype via ROS-HMGB1-TLR4 axis and inhibition of tumor growth and metastasis. Theranostics. 2024;14(6):2605–2621. 38646639 10.7150/thno.92672 PMC11024855

[132] Vanmeerbeek I, Naulaerts S, Sprooten J, Laureano RS, Govaerts J, Trotta R, Pretto S, Zhao S, Cafarello ST, Verelst J, et al. Targeting conserved TIM3^+^VISTA^+^ tumor-associated macrophages overcomes resistance to cancer immunotherapy. Sci Adv. 2024;10(29): Article eadm8660. 39028818 10.1126/sciadv.adm8660 PMC11259173

[133] Choi Y, Kim SA, Jung H, Kim E, Kim YK, Kim S, Kim J, Lee Y, Jo MK, Woo J, et al. Novel insights into paclitaxel’s role on tumor-associated macrophages in enhancing PD-1 blockade in breast cancer treatment. J Immunother Cancer. 2024;12(7): Article e008864. 39009452 10.1136/jitc-2024-008864 PMC11253755

[134] Cullis J, Siolas D, Avanzi A, Barui S, Maitra A, Bar-Sagi D. Macropinocytosis of nab-paclitaxel drives macrophage activation in pancreatic cancer. Cancer Immunol Res. 2017;5(3):182–190. 28108630 10.1158/2326-6066.CIR-16-0125 PMC5570452

[135] Leonard F, Curtis LT, Ware MJ, Nosrat T, Liu X, Yokoi K, Frieboes HB, Godin B. Macrophage polarization contributes to the anti-tumoral efficacy of mesoporous nanovectors loaded with albumin-bound paclitaxel. Front Immunol. 2017;8:693. 28670313 10.3389/fimmu.2017.00693 PMC5472662

[136] Tang W, Yang J, Yuan Y, Zhao Z, Lian Z, Liang G. Paclitaxel nanoparticle awakens immune system to fight against cancer. Nanoscale. 2017;9(19):6529–6536. 28466929 10.1039/c6nr09895a

[137] Peng J, Yang Q, Jiang H, Wang Y, Liu Q, Xiao Y, Qian Z. Amplifying cancer chemoimmunotherapy through F4/80^+^CD11c^+^ DC-like macrophages induced by micellar docetaxel together with a TLR7/8 nanoagonist. Nano Today. 2024;54: Article 102087.

[138] Cao X, Li B, Chen J, Dang J, Chen S, Gunes EG, Xu B, Tian L, Muend S, Raoof M, et al. Effect of cabazitaxel on macrophages improves CD47-targeted immunotherapy for triple-negative breast cancer. J Immunother Cancer. 2021;9(3): Article e002022. 33753567 10.1136/jitc-2020-002022 PMC7986678

[139] Millrud CR, Mehmeti M, Leandersson K. Docetaxel promotes the generation of anti-tumorigenic human macrophages. Exp Cell Res. 2018;362(2):525–531. 29269075 10.1016/j.yexcr.2017.12.018

[140] Roux C, Jafari SM, Shinde R, Duncan G, Cescon DW, Silvester J, Chu MF, Hodgson K, Berger T, Wakeham A, et al. Reactive oxygen species modulate macrophage immunosuppressive phenotype through the up-regulation of PD-L1. Proc Natl Acad Sci USA. 2019;116(10):4326–4335. 30770442 10.1073/pnas.1819473116 PMC6410837

[141] Sekiguchi F, Domoto R, Nakashima K, Yamasoba D, Yamanishi H, Tsubota M, Wake H, Nishibori M, Kawabata A. Paclitaxel-induced HMGB1 release from macrophages and its implication for peripheral neuropathy in mice: Evidence for a neuroimmune crosstalk. Neuropharmacology. 2018;141:201–213. 30179591 10.1016/j.neuropharm.2018.08.040

[142] Bald T, Krummel MF, Smyth MJ, Barry KC. The NK cell-cancer cycle: Advances and new challenges in NK cell-based immunotherapies. Nat Immunol. 2020;21(8):835–847. 32690952 10.1038/s41590-020-0728-z PMC8406687

[143] Daher M, Melo Garcia L, Li Y, Rezvani K. CAR-NK cells: The next wave of cellular therapy for cancer. Clin Transl Immunol. 2021;10(4): Article e1274. 10.1002/cti2.1274 PMC8080297 33959279

[144] Cifaldi L, Locatelli F, Marasco E, Moretta L, Pistoia V. Boosting natural killer cell-based immunotherapy with anticancer drugs: A perspective. Trends Mol Med. 2017;23(12):1156–1175. 29133133 10.1016/j.molmed.2017.10.002

[145] Law KS, Chen H-C, Liao S-K. Non-cytotoxic and sublethal paclitaxel treatment potentiates the sensitivity of cultured ovarian tumor SKOV-3 cells to lysis by lymphokine-activated killer cells. Anticancer Res. 2007;27(2):841–850. 17465210

[146] Di Modica M, Sfondrini L, Regondi V, Varchetta S, Oliviero B, Mariani G, Bianchi GV, Generali D, Balsari A, Triulzi T, et al. Taxanes enhance trastuzumab-mediated ADCC on tumor cells through NKG2D-mediated NK cell recognition. Oncotarget. 2015;7(1):255–265. 10.18632/oncotarget.6353 PMC4807996 26595802

[147] Acebes-Huerta A, Lorenzo-Herrero S, Folgueras AR, Huergo-Zapico L, Lopez-Larrea C, López-Soto A, Gonzalez S. Drug-induced hyperploidy stimulates an antitumor NK cell response mediated by NKG2D and DNAM-1 receptors. Oncoimmunology. 2016;5(2): Article e1074378. 27057443 10.1080/2162402X.2015.1074378 PMC4801427

[148] Tang M, Gao S, Zhang L, Liu B, Li J, Wang Z, Zhang W. Docetaxel suppresses immunotherapy efficacy of natural killer cells toward castration-resistant prostate cancer cells via altering androgen receptor-lectin-like transcript 1 signals. Prostate. 2020;80(10):742–752. 32449811 10.1002/pros.23988

[149] Loubani O, Hoskin DW. Paclitaxel inhibits natural killer cell binding to target cells by down-regulating adhesion molecule expression. Anticancer Res. 2005;25(2A):735–741. 15868904

[150] Kubo M, Morisaki T, Matsumoto K, Tasaki A, Yamanaka N, Nakashima H, Kuroki H, Nakamura K, Nakamura M, Katano M. Paclitaxel probably enhances cytotoxicity of natural killer cells against breast carcinoma cells by increasing perforin production. Cancer Immunol Immunother. 2005;54(5):468–476. 15592829 10.1007/s00262-004-0617-6 PMC11033023

[151] Ostrand-Rosenberg S, Sinha P, Beury DW, Clements VK. Cross-talk between myeloid-derived suppressor cells (MDSC), macrophages, and dendritic cells enhances tumor-induced immune suppression. Semin Cancer Biol. 2012;22(4):275–281. 22313874 10.1016/j.semcancer.2012.01.011 PMC3701942

[152] Yang EL, Sun ZJ. Nanomedicine targeting myeloid-derived suppressor cells enhances anti-tumor immunity. Adv Healthc Mater. 2024;13(9): Article e2303294. 38288864 10.1002/adhm.202303294

[153] Michels T, Shurin GV, Naiditch H, Sevko A, Umansky V, Shurin MR. Paclitaxel promotes differentiation of myeloid-derived suppressor cells into dendritic cells in vitro in a TLR4-independent manner. J Immunotoxicol. 2012;9(3):292–300. 22283566 10.3109/1547691X.2011.642418 PMC3386478

[154] Kodumudi KN, Woan K, Gilvary DL, Sahakian E, Wei S, Djeu JY. A novel chemoimmunomodulating property of docetaxel: Suppression of myeloid-derived suppressor cells in tumor bearers. Clin Cancer Res. 2010;16(18):4583–4594. 20702612 10.1158/1078-0432.CCR-10-0733 PMC3874864

[155] Sevko A, Michels T, Vrohlings M, Umansky L, Beckhove P, Kato M, Shurin GV, Shurin MR, Umansky V. Antitumor effect of paclitaxel is mediated by inhibition of myeloid-derived suppressor cells and chronic inflammation in the spontaneous melanoma model. J Immunol. 2013;190(5):2464–2471. 23359505 10.4049/jimmunol.1202781 PMC3578135

[156] Vankerckhoven A, Baert T, Riva M, De Bruyn C, Thirion G, Vandenbrande K, Ceusters J, Vergote I, Coosemans A. Type of chemotherapy has substantial effects on the immune system in ovarian cancer. Transl Oncol. 2021;14(6): Article 101076. 33770618 10.1016/j.tranon.2021.101076 PMC8022256

[157] Gebhardt C, Simon SCS, Weber R, Gries M, Mun DH, Reinhard R, Holland-Letz T, Umansky V, Utikal J. Potential therapeutic effect of low-dose paclitaxel in melanoma patients resistant to immune checkpoint blockade: A pilot study. Cell Immunol. 2021;360: Article 104274. 33383383 10.1016/j.cellimm.2020.104274

[158] Meng F, Wang J, He Y, Cresswell GM, Lanman NA, Lyle LT, Ratliff TL, Yeo Y. A single local delivery of paclitaxel and nucleic acids via an immunoactive polymer eliminates tumors and induces antitumor immunity. Proc Natl Acad Sci USA. 2022;119(22): Article e2122595119. 35609195 10.1073/pnas.2122595119 PMC9295735

[159] Maulhardt H, Marin A, Hesseltine H, diZerega G. Submicron particle docetaxel intratumoral injection in combination with anti-mCTLA-4 into 4T1-Luc orthotopic implants reduces primary tumor and metastatic pulmonary lesions. Med Oncol. 2021;38(9):106. 34331595 10.1007/s12032-021-01555-1 PMC8325653

[160] Zhang R, Wan Y, Lv H, Li F, Lee CS. DTX@VTX NPs synergy PD-L1 immune checkpoint nanoinhibitor to reshape immunosuppressive tumor microenvironment for enhancing chemo-immunotherapy. J Mater Chem B. 2021;9(36):7544–7556. 34551052 10.1039/d1tb00269d

[161] Kadiyala P, Li D, Nuñez FM, Altshuler D, Doherty R, Kuai R, Yu M, Kamran N, Edwards M, Moon JJ, et al. High-density lipoprotein-mimicking nanodiscs for chemo-immunotherapy against glioblastoma multiforme. ACS Nano. 2019;13(2):1365–1384. 30721028 10.1021/acsnano.8b06842 PMC6484828

[162] Kim J, Thomas SN. Opportunities for nitric oxide in potentiating cancer immunotherapy. Pharmacol Rev. 2022;74(4):1146–1175. 36180108 10.1124/pharmrev.121.000500 PMC9553106

[163] Li X, Yuan K, Yin Y, Tian Y, Guo Z, Qin Z, Zeng X. Docetaxel-loaded electrospun nanofibrous mats for local chemotherapy targeting positive surgical margins in prostate cancer. Mol Pharm. 2025;22(4):2213–2223. 40073383 10.1021/acs.molpharmaceut.4c01440

[164] Gu Z, Wang Q, Shi Y, Huang Y, Zhang J, Zhang X, Lin G. Nanotechnology-mediated immunochemotherapy combined with docetaxel and PD-L1 antibody increase therapeutic effects and decrease systemic toxicity. J Control Release. 2018;286:369–380. 30096401 10.1016/j.jconrel.2018.08.011

[165] Hu N, Xue H, Zhang T, Fan Y, Guo F, Li Z, Huo M, Guan X, Chen G. Harnessing PD-1 cell membrane-coated paclitaxel dimer nanoparticles for potentiated chemoimmunotherapy. Biomed Pharmacother. 2024;174: Article 116482. 38520866 10.1016/j.biopha.2024.116482

[166] Li Z, Pei Q, Zhao M, Xie Z, Zheng M. Self-carrier nanoparticles for delivery of paclitaxel and IDO inhibitor to boost antitumor chemo-immunotherapy. Adv Funct Mater. 2024;34(30):2312500.

[167] Luo K, Lian Y, Zhang M, Yu H, Wang G, Li J. Charge convertible biomimetic micellar nanoparticles for enhanced melanoma-targeted therapy through tumor cells and tumor-associated macrophages dual chemotherapy with IDO immunotherapy. Chem Eng J. 2021;412: Article 128659.

[168] Hu Z, Zheng B, Xu J, Gao S, Lu W. An albumin-bound drug conjugate of paclitaxel and indoleamine-2,3-dioxygenase inhibitor for enhanced cancer chemo-immunotherapy. Nanotechnology. 2020;31(29): Article 295101. 32203949 10.1088/1361-6528/ab824d

[169] Lang T, Zheng Z, Huang X, Liu Y, Zhai Y, Zhang P, Li Y, Yin Q. Ternary regulation of tumor microenvironment by heparanase-sensitive micelle-loaded monocytes improves chemo-immunotherapy of metastatic breast cancer. Adv Funct Mater. 2020;31(10): Article 2007402.

[170] Qiao H, Chen X, Chen E, Zhang J, Huang D, Yang D, Ding Y, Qian H, Feijen J, Chen W. Folated pH-degradable nanogels for the simultaneous delivery of docetaxel and an IDO1-inhibitor in enhancing cancer chemo-immunotherapy. Biomater Sci. 2019;7(7):2749–2758. 30997445 10.1039/c9bm00324j

[171] Li M, Lu L, Guo Y, Fu J, Zhang Z, Li P, Guo Y, Han M, Wang X. Self-assembly of paclitaxel derivative and fructose as a potent inducer of immunogenic cell death to enhance cancer immunotherapy. Mater Today Bio. 2025;32: Article 101793. 10.1016/j.mtbio.2025.101793 PMC12059333 40343162

[172] Guo B, Qu Y, Sun Y, Zhao S, Yuan J, Zhang P, Zhong Z, Meng F. Co-delivery of gemcitabine and paclitaxel plus NanoCpG empowers chemoimmunotherapy of postoperative “cold” triple-negative breast cancer. Bioact Mater. 2023;25:61–72. 36733927 10.1016/j.bioactmat.2023.01.014 PMC9879764

[173] Lim C, Hwang D, Yazdimamaghani M, Atkins HM, Hyun H, Shin Y, Ramsey JD, Radler PD, Mott KR, Perou CM, et al. High-dose paclitaxel and its combination with CSF1R inhibitor in polymeric micelles for chemoimmunotherapy of triple negative breast cancer. Nano Today. 2023;51: Article 101884. 37484164 10.1016/j.nantod.2023.101884 PMC10357922

[174] Chen L, Hu M, Yan HM, Ding XQ, Yang QX, Wang L, Pan H. Dual-targeted albumin nanoparticles for the co-delivery of low-dose paclitaxel and PCSK9 inhibitor in melanoma treatment. Mater Today Bio. 2025;35: Article 102329. 10.1016/j.mtbio.2025.102329 PMC12510023 41080729

[175] Hu Q, Shang L, Wang M, Tu K, Hu M, Yu Y, Xu M, Kong L, Guo Y, Zhang Z. Co-delivery of paclitaxel and interleukin-12 regulating tumor microenvironment for cancer immunochemotherapy. Adv Healthc Mater. 2020;9(10): Article e1901858. 32348030 10.1002/adhm.201901858

[176] Cao L, Zeng Q, Xu C, Shi S, Zhang Z, Sun X. Enhanced antitumor response mediated by the codelivery of paclitaxel and adenoviral vector expressing IL-12. Mol Pharm. 2013;10(5):1804–1814. 23534449 10.1021/mp300602j

[177] Qian X, Yang H, Ye Z, Gao B, Qian Z, Ding Y, Mao Z, Du Y, Wang W. Celecoxib augments paclitaxel-induced immunogenic cell death in triple-negative breast cancer. ACS Nano. 2024;18(24):15864–15877. 38829727 10.1021/acsnano.4c02947

[178] Shi X, Shu L, Qiao Y, Yao J, Xie H, Zhou L, Wang H, Zheng S. Synergistic nanoassemblies constructed from a STAT3 inhibitor and a cabazitaxel prodrug with enhanced cancer chemo-immunotherapy. Mater today Nano. 2022;17: Article 100155.

[179] Chen Q, Zhang L, Li L, Tan M, Liu W, Liu S, Xie Z, Zhang W, Wang Z, Cao Y, et al. Cancer cell membrane-coated nanoparticles for bimodal imaging-guided photothermal therapy and docetaxel-enhanced immunotherapy against cancer. J Nanobiotechnology. 2021;19(1):449. 34952587 10.1186/s12951-021-01202-x PMC8710014

[180] Peng J, Yang Q, Xiao Y, Shi K, Liu Q, Hao Y, Yang F, Han R, Qian Z. Tumor microenvironment responsive drug-dye-peptide nanoassembly for enhanced tumor-targeting, penetration, and photo-chemo-immunotherapy. Adv Funct Mater. 2019;29(19): Article 1900004.

[181] Chen L, Zhou L, Wang C, Han Y, Lu Y, Liu J, Hu X, Yao T, Lin Y, Liang S, et al. Tumor-targeted drug and CpG delivery system for phototherapy and docetaxel-enhanced immunotherapy with polarization toward M1-type macrophages on triple negative breast cancers. Adv Mater. 2019;31(52): Article e1904997. 31721331 10.1002/adma.201904997

[182] Kang RH, Rasoulianboroujeni M, Kianpour M, Repp L, Ponik SM, Kwon GS. Precise ratiometric drug delivery for the treatment of triple-negative breast cancer. ACS Nano. 2025;19(47):40456–40472. 41258901 10.1021/acsnano.5c13083 PMC12676740

[183] Cho KJ, Cho YE, Kim J. Locoregional lymphatic delivery systems using nanoparticles and hydrogels for anticancer immunotherapy. Pharmaceutics. 2022;14(12):2752. 36559246 10.3390/pharmaceutics14122752 PMC9788085

[184] Kim J, Archer PA, Thomas SN. Innovations in lymph node targeting nanocarriers. Semin Immunol. 2021;56: Article 101534. 34836772 10.1016/j.smim.2021.101534 PMC8792237

[185] Han CY, Choi S-H, Chi S-H, Hong JH, Cho Y-E, Kim J. Nano-fluorescence imaging: Advancing lymphatic disease diagnosis and monitoring. Nano Converg. 2024;11(1):53. 39661218 10.1186/s40580-024-00462-1 PMC11635084

[186] Francis DM, Manspeaker MP, Schudel A, Sestito LF, O’Melia MJ, Kissick HT, Pollack BP, Waller EK, Thomas SN. Blockade of immune checkpoints in lymph nodes through locoregional delivery augments cancer immunotherapy. Sci Transl Med. 2020;12(563): Article eaay3575. 32998971 10.1126/scitranslmed.aay3575 PMC8377700

[187] Manspeaker MP, O’Melia MJ, Thomas SN. Elicitation of stem-like CD8^+^ T cell responses via lymph node-targeted chemoimmunotherapy evokes systemic tumor control. J Immunother Cancer. 2022;10(9): Article e005079. 36100312 10.1136/jitc-2022-005079 PMC9472119

[188] Reichel D, Tripathi M, Perez JM. Biological effects of nanoparticles on macrophage polarization in the tumor microenvironment. Nanotheranostics. 2019;3(1):66–88. 30662824 10.7150/ntno.30052 PMC6328304

[189] Yang W, Frickenstein AN, Sheth V, Holden A, Mettenbrink EM, Wang L, Woodward AA, Joo BS, Butterfield SK, Donahue ND, et al. Controlling nanoparticle uptake in innate immune cells with heparosan polysaccharides. Nano Lett. 2022;22(17):7119–7128. 36048773 10.1021/acs.nanolett.2c02226 PMC9486251

[190] Kumar S, Anselmo AC, Banerjee A, Zakrewsky M, Mitragotri S. Shape and size-dependent immune response to antigen-carrying nanoparticles. J Control Release. 2015;220(Pt A):141–148. 26437263 10.1016/j.jconrel.2015.09.069

[191] Alishekevitz D, Gingis-Velitski S, Kaidar-Person O, Gutter-Kapon L, Scherer SD, Raviv Z, Merquiol E, Ben-Nun Y, Miller V, Rachman-Tzemah C, et al. Macrophage-induced lymphangiogenesis and metastasis following paclitaxel chemotherapy is regulated by VEGFR3. Cell Rep. 2016;17(5):1344–1356. 27783948 10.1016/j.celrep.2016.09.083 PMC5098117

[192] Harris AR, Perez MJ, Munson JM. Docetaxel facilitates lymphatic-tumor crosstalk to promote lymphangiogenesis and cancer progression. BMC Cancer. 2018;18(1):718. 29976154 10.1186/s12885-018-4619-8 PMC6034223

[193] Li Y, Wu X, Fang D, Luo Y. Informing immunotherapy with multi-omics driven machine learning. NPJ Digit Med. 2024;7(1):67. 38486092 10.1038/s41746-024-01043-6 PMC10940614

